# Complex cytogeographical patterns reveal a dynamic tetraploid–octoploid contact zone

**DOI:** 10.1093/aobpla/ply012

**Published:** 2018-02-14

**Authors:** Mariana Castro, Sílvia Castro, Albano Figueiredo, Brian Husband, João Loureiro

**Affiliations:** 1 CFE, Centre for Functional Ecology, Department of Life Sciences, University of Coimbra, Calçada Martim de Freitas, Coimbra, Portugal; 2 Botanic Garden of the University of Coimbra, Calçada Martim de Freitas, Coimbra, Portugal; 3 CEGOT, Departamento de Geografia e Turismo, Faculdade de Letras, Universidade de Coimbra, Largo da Porta Férrea, Coimbra, Portugal; 4 Department of Integrative Biology, University of Guelph, Guelph, ON, Canada

**Keywords:** Contact zone, distribution patterns, *Gladiolus communis*, hexaploid, hybridization, niche modelling, niche overlapping, octoploid, tetraploid

## Abstract

The distribution of cytotypes in mixed-ploidy species is crucial for evaluating ecological processes involved in the establishment and evolution of polyploid taxa. Here, we use flow cytometry and chromosome counts to explore cytotype diversity and distributions within a tetraploid–octoploid contact zone. We then use niche modelling and ploidy seed screening to assess the roles of niche differentiation among cytotypes and reproductive interactions, respectively, in promoting cytotype coexistence. Two cytotypes, tetraploids and octoploids, were dominant within the contact zone. They were most often distributed parapatrically or allopatrically, resulting in high geographic isolation. Still, 16.7 % of localities comprised two or more cytotypes, including the intermediate hexaploid cytotype. Tetraploids and octoploids had high environmental niche overlap and associated with similar climatic environments, suggesting they have similar ecological requirements. Given the geographical separation and habitat similarity among cytotypes, mixed-ploidy populations may be transitional and subject to the forces of minority cytotype exclusion which lead to pure-ploidy populations. However, seed ploidy analysis suggests that strong reproductive barriers may enforce assortative mating which favours stable cytotype coexistence. High cytogenetic diversity detected in the field suggests that unreduced gamete formation and hybridization events seem frequent in the studied polyploid complex and might be involved with the recurrent polyploid formation, governing, as well, the gene flow between cytogenetic entities.

## Teaser

Cytotype distribution patterns are crucial for evaluating ecological processes involved in polyploid establishment and evolution. We explore cytotype diversity and distribution in contact zones of a tetraploid–octoploid plant. Complex contact zones and high cytogenetic diversity in natural populations were observed. Tetraploids and octoploids show geographical separation and habitat similarity suggesting that mixed-ploidy populations are subject to frequency-dependent selection, although the existence of reproductive barriers may enable cytotype coexistence. The detection of hexaploids in different points of the distribution indicates that unreduced gamete formation and hybridization occur and might be involved with recurrent polyploid formation and gene flow between cytotypes.

## Introduction

Polyploidization, the duplication of complete chromosome sets, is widely considered an important mechanism of plant evolution (Soltis and Soltis 1999; Jiao *et al.* 2011) and sympatric speciation (Otto and Whitton 2000; Soltis *et al.* 2010). Based on recent molecular and fossil studies, polyploidy has been linked with radiations in species diversity throughout evolutionary history (Soltis *et al.* 2009) and associated with 15 % of speciation events in extant angiosperms (Wood *et al.* 2009). Consequently, polyploidy is pervasive in flowering plants. The standing incidence of polyploid species is estimated at 35 % (Wood *et al.* 2009), with higher values being observed in specific geographic regions such as the Mediterranean basin (ranging between 37 and 47 %; Marques *et al.* 2017) and the Arctic region (69 and 87 %; Brochmann *et al.* 2004).

The geographic distribution of polyploids is useful for inferring mechanisms of polyploid evolution, coexistence and divergence. The spatial arrangement of cytotypes *in situ* is the result of several interacting processes operating in natural populations including formation and migration; ecological preferences, and competitive and dispersal abilities; and reproductive interactions, among others (Petit *et al.* 1999; Levin 2002; Lexer and van Loo 2006). Cytotype distributions can be characterized as sympatric, parapatric or allopatric depending on whether the different cytotypes grow intermixed, adjacent or disjunct, respectively (Petit *et al.* 1999; and illustrated in Fig. 2 of Mallet *et al.* 2009, which can be applied to polyploid complexes). Theoretical models predict that within zones of sympatry, mixed-ploidy populations are expected to be rare and evolutionarily unstable because frequency-dependent selection will drive the exclusion of the minority cytotype (Levin 1975; Rodriguez 1996; Husband and Schemske 2000). Still, numerous studies have documented mixed-ploidy populations (reviewed in Husband *et al.* 2013; and examples below). The presence of multiple cytotypes in the same population can reflect either a transitory stage, in which neopolyploids are recurrently formed, or a persistent stage such as when cytotypes are ecologically and reproductively isolated on a small spatial scale (e.g. Kolář *et al.* 2009; Jersáková *et al.* 2010). In this context, assessing the distribution of cytotypes within and among natural populations is crucial to build and test hypotheses that account for the successful establishment of polyploids.

Contact zones, areas with two or more cytotypes growing in close proximity, are thus considered natural laboratories within which to study evolutionary transitions through polyploidy. In recent years, an increasing number of studies have provided insights into ploidy-mediated processes occurring in contact zones (e.g. Husband *et al.* 2013; Ramsey and Ramsey 2014). Significant advances in this field have been largely fuelled by the ability to rapidly and easily screen thousands of individuals using flow cytometry (Kron *et al.* 2007). This approach has resulted in a proliferation of cytogeographical studies (e.g. Baack 2004; Kolář *et al.* 2009; Ståhlberg 2009; Trávníček *et al.* 2010; Castro *et al.* 2012; Zozomová-Lihová *et al.* 2015; Wefferling *et al.* 2017; reviewed in Ramsey and Ramsey 2014), which detect extensive cytogenetic diversity and, in several cases, occurrence of mixed-ploidy populations (e.g. Baack 2004; Kolář *et al.* 2009; Trávníček *et al.* 2010; Castro *et al.* 2012; Zozomová-Lihová *et al.* 2015; Wefferling *et al.* 2017), rare cytotypes (e.g. Kolář *et al.* 2009; Trávníček *et al.* 2010), production of unreduced gametes (e.g. Maceira *et al.* 1992; Burton and Husband 2001; Ramsey 2007; Castro *et al.* 2016a) or recurrent occurrence of gene flow (e.g. Husband 2004; Kolář *et al.* 2009; Castro *et al.* 2011). Particularly interesting are polyploid complexes with higher ploidies, such as diploid–hexaploid (e.g. *Aster amellus*; Castro *et al.* 2012) or tetraploid–octoploid complexes (e.g. *Gymnadenia conopsea*; Jersáková *et al.* 2010), that can produce even-ploidy hybrids, which are potentially more stable and lead to highly dynamic contact zones. Regardless of the increasing number of studies at contact zones, the available information is still scarce and insufficient for many plant groups and regions (Soltis *et al.* 2010, 2016; Marques *et al.* 2017).

*Gladiolus communis* is a Mediterranean polyploid complex with high morphological variation (Alonso and Crespo 2010). Multiple ploidy levels have been described for the complex, namely tetraploids (2*n* = 4*x* = 60 chromosomes; Fernandes *et al.* 1948; Fernandes 1950; Nilsson and Lassen 1971; Queirós 1980; Fernández *et al.* 1985) and octoploids (2*n* = 8*x* = 120; Fernandes and Queirós 1971; Löve and Kjellqvist 1973; Queirós 1980), although hexaploids (2*n* = 6*x* = 90) and duodecaploids (2*n* = 12*x* = 180) have also been occasionally reported in the Mediterranean basin (Darlington and Wylie 1955). The Iberian Peninsula seems to harbour this diversity (Fernandes *et al.* 1948; Fernandes and Queirós 1971; Queirós 1979) and areas of close contact between tetraploids and octoploids have been detected, for example, in calcareous regions from Central Portugal (Castro *et al.* 2016b). Occasionally, *G. communis* grows with another congeneric species, namely *G. italicus*, which, in the Iberian Peninsula, is represented by duodecaploid individuals (Queirós 1979; Pérez and Pastor Díaz 1994; although octoploids have also been described in the Mediterranean basin, e.g. Susnik and Lovka 1973; Strid and Franzen 1981; van Raamsdonk and De Vries 1989; Kamari *et al.* 2001). The high morphological variation of the group has led taxonomists to accept multiple taxonomic entities within the *G. communis* complex (e.g. Gussone 1832; van Raamsdonk and De Vries 1989), although morphologically intermediate forms are found in natural populations, and many characters used to distinguish each taxon are extremely variable and largely overlap, even within populations (Hamilton 1980; revised in Alonso and Crespo 2010). Consequently, recent morphological reviews and preliminary molecular analyses failed to support the previous taxonomic delimitations and the species is currently accepted as a complex formed by three ploidy levels (Buchanan 2008; Alonso and Crespo 2010). Regardless of the variability detected in the species, nothing is known about the role of genome duplications generating diversity within this polyploid complex. Exploring cytotype diversity and distribution patterns, especially at contact zones, is thus crucial to understand ecological processes, such as ecological preferences and reproductive interactions, driving current diversity patterns at natural contact zones.

In this study, we explore in detail the cytotype diversity and distribution patterns in a tetraploid–octoploid *G. communis* contact zone. In particular, we pose the following specific questions: (i) what are the dominant cytotypes and how are they distributed in the contact zone? (ii) do cytotypes coexist and at which spatial scale? (iii) is coexistence facilitated by differences in environmental associations between cytotypes? And finally, (iv) is there evidence for the production of unreduced gametes and/or cytotype hybridization? To address our questions, cytotype diversity was studied at several spatial scales, namely (i) across the contact zone, to characterize the most dominant cytotypes and their environmental preferences within areas of contact; (ii) within mixed-ploidy populations, to measure microhabitat segregation; and (iii) among offspring from plants in pure- and mixed-ploidy populations, to detect cytotype diversity at early stages. Flow cytometric analyses complemented with chromosome counts were used to assess ploidy levels of all the sampled individuals. The reproductive success of pure- and mixed-ploidy populations was also quantified in natural conditions to depict fitness differences between cytotypes. The spatial arrangement of cytotypes in the contact zone was analysed with niche modelling tools to determine if differences in environmental requirements could explain cytotype distribution. If cytotypes differ in environmental requirements, we expect a mosaic contact zone with tetraploids and octoploids fairly isolated within a given spatial scale and with plants growing in different habitats or microhabitats. If no environmental differences are observed, we expect a tension zone where sympatric cytotype co-occurrence is possible, where intermediate cytotypes are detected and where other processes such as reproductive barriers, competition or dispersal abilities are expected to play major roles in driving distribution patterns.

## Methods

### Study system and studied region

*Gladiolus communis* is a perennial species that is widespread on the Iberian Peninsula and throughout the Mediterranean basin. The species produces an ovoid bulb, relatively thick roots, a cylindrical glabrous stem and linear leaves with typical parallel ribs. The pink bisexual flowers are zygomorphic and usually grouped in one spiked inflorescence per individual. A second *Gladiolus* species, *G. italicus*, is found on the Iberian Peninsula and occurs in sympatry with *G. communis* in some places. Although very similar morphologically, these two species are easily distinguished based on inflorescence architecture, anther and filament lengths, and seed morphology. *Gladiolus communis* has a unilateral inflorescence, anthers equalling or shorter than the filaments, and broadly winged seeds, while *G. italicus* usually has a weakly distichous inflorescence, anthers longer than the filaments, and polyhedric apterous seeds (Hamilton 1980; Alonso and Crespo 2010).

In the Iberian Peninsula, *G. communis* is recognized as a polyploid complex comprising tetraploids (2*n* = 4*x* = 60 chromosomes), hexaploids (2*n* = 6*x* = 90) and octoploids (2*n* = 8*x* = 120) (e.g. Fernandes *et al.* 1948; Fernandes and Queirós 1971; Alonso and Crespo 2010) with duodecaploids being described elsewhere in the Mediterranean region (Darlington and Wylie 1955). The high morphological resemblance among *G. communis* cytotypes (Alonso and Crespo 2010; Cantor and Tolety 2011) suggests a putative autopolyploid origin. The species is common in the calcareous regions from Central Portugal, where preliminary field sampling revealed the presence of tetraploid and octoploid populations growing in close proximity. This study focused on this contact zone, an area extending from 39.3° to 40.6° in latitude, and from 7.8° to 9.4° in longitude. This territory is dominated by calcareous rocks and presents a Mediterranean climate that exhibits a strong influence from the Atlantic Ocean, an attribute identified on the significant values of annual precipitation (1000–1300 mm). However, the dominance of poor soils determines a low water storage capacity, which, combined with a long and hot summer, determines the dominance of evergreen vegetation. Allied to such climatic conditions, human pressure contributed to current dominance of shrubby communities in the landscape, and constrained forests (evergreen and semi-deciduous) to very small patches, favouring the wide presence of open habitats. These open habitats are also characterized by the presence of limestone outcrops exposed to stressful ecological conditions that limit the installation of higher vegetation covers.

Although not exhaustive, additional sampling was extended beyond this area to determine the dominant cytotypes within the species. Also, because *G. communis* coexist with *G. italicus*, hybridization might occur and generate additional cytogenetic diversity, the duodecaploid *G. italicus* (Queirós 1979; Pérez and Pastor Díaz 1994) was also sampled whenever growing with *G. communis*.

### Field sampling

Field collections were carried out during the flowering and fruiting seasons (mid-April to July) of *G. communis* from 2012 to 2015. Individual plants or clusters of plants were easily detected when blooming because of the tall inflorescences growing above the remaining vegetation. We sampled 81 populations across the contact zone where both tetraploid and octoploid cytotypes have been previously detected in close proximity. An additional group of 27 populations covering the western distribution of the species around the contact zone was also sampled to depict the dominant cytotypes **[see** **Supporting Information—Table S1****]**. In each of the 108 populations, we collected ~3 cm^2^ of fresh leaf of up to 53 individuals of *G. communis* (with an average of 20 individuals per locality, excluding two particularly large localities where more intensive sampling was done, with 106 and 454 plants being screened), and of *G. italicus* whenever detected growing with *G. communis* (up to 32 individuals, averaging 13 plants per locality). The sampled individuals were randomly selected, covering the extension of the population. Leaves were stored in labelled hermetic bags and maintained at 4 °C for later flow cytometric analysis (see section *Genome size and DNA ploidy level estimates*). Geographic coordinates of the population were recorded. Bulbs of nine localities identified in preliminary surveys as DNA tetraploid, DNA hexaploid and DNA octoploid populations (Castro *et al.* 2016b) were also collected, potted and maintained at the common garden for chromosome counts (see section *Chromosome counts*).

In addition, we sampled in mixed-ploidy populations more intensively to test for microhabitat segregation. Three mixed-ploidy populations containing tetraploids, hexaploids and/or octoploids were revisited and all adult, individuals (both vegetative and reproductive individuals) were mapped with *x/y* coordinates, tagged and sampled for ploidy level analyses using flow cytometry (see section *Genome size and DNA ploidy level estimates*). To delimit the clusters of plants growing in sympatry, screenings for *Gladiolus* plants were made around a radius of over 150 m around the cluster of plants initially detected or until an anthropogenic or natural barrier was observed. Additional mixed-ploidy populations were not sampled because they were disturbed by grazing or human activities.

Finally, we screened offspring from plants in pure- and mixed-ploidy populations to examine the production of unreduced gametes and/or hybridization events by the detection of rare cytotypes that might not reach the adult stage. For this, four tetraploid, two hexaploid, four octoploid and one mixed tetraploid–octoploid populations were revisited and individual plants with known ploidy were sampled to determine reproductive success and screen ploidy of the seeds (see section *Reproductive success in natural populations*).

### Chromosome counts

Chromosome counts were used to calibrate genome size estimates, obtained using flow cytometry, to a given ploidy level. For this, the plants grown from bulbs collected in the selected natural populations and maintained in the common garden were used simultaneously for genome size estimates and chromosome counting. For chromosome counts, we followed the protocol of Goldblatt and Takei (1993), with some adjustments. Briefly, actively growing root tips were harvested and pretreated in 0.002 M aqueous 8-hydroquinoline at room temperature for 1630 h, and fixed in 95 % ethanol and glacial acetic acid (in a ratio of 3:1) for at least 48 h at 4 °C. Roots tips were hydrolysed in 1 M hydrogen chloride at 60 °C in a sand bath for 40 min, submerged in Schiff reagent (Greilhuber and Ebert 1994) for 1330 h, washed in sulphur water for three periods of 10 min and finally squashed under a glass cover in a drop of acetic orcein 2 %. Chromosome spreads were observed using a Nikon Eclipse 80i light microscope and photographed using a Nikon Plan Apo VC 100×/1.40 oil-immersion lens with a Q Imaging Retiga 2000R Fast 1394 digital camera and Q-Capture Pro v.7 software. A total of 40 individuals from nine populations were used to access chromosome number and genome size: 4*x*—populations MC147 (*n* = 10 individuals), MC193 (*n* = 1), MC195 (*n* = 4), MC201 (*n* = 1) and MC212 (*n* = 2); 6*x*—population MC211 (*n* = 4); 8*x*—populations MC032 (*n* = 8), MC143 (*n* = 3), MC190 (*n* = 4), MC193 (*n* = 2) and MC201 (*n* = 1) **[see** **Supporting Information—Table S1****]**.

### Genome size and DNA ploidy level estimates

To estimate genome size and DNA ploidy levels, fresh leaves collected in natural populations were analysed using flow cytometry. Nuclear suspensions were prepared following Galbraith *et al.* (1983) by chopping the plant material of the sampled species together with leaf tissue of an internal reference standard. In the case of *Gladiolus* nuclear suspensions, 100 mg of leaf tissue or 2–5 seeds were co-chopped with 50 mg of leaf of *Solanum lycopersicum* ‘Stupické’ (2C = 1.96 pg; Doležel *et al.* 1992) or *Pisum sativum* ‘Ctirad’ (2C = 9.09 pg; Doležel *et al.* 1998). *Solanum lycopersicum* was used as the internal standard in most cases, except when unavailable, with *P. sativum* being used in those situations. Sample and standard were co-chopped in 1 mL of WPB buffer (WPB: 0.2 M Tris–HCl, 4 mM MgCl_2_·6H_2_O, 1 % Triton X-100, 2 mM EDTA Na_2_·2H_2_O, 86 mM NaCl, 10 mM metabisulfite, 1 % PVP-10, pH adjusted to 7.5 and stored at 4 °C; Loureiro *et al.* 2007) using a razor blade. The resulting nuclear suspension was filtered through a 50 µm nylon filter and 50 µg mL^−1^ propidium iodide (Fluka, Buchs, Switzerland) and 50 µg mL^−1^ RNAse (Fluka) were added to the sample, to stain the DNA and avoid staining of double-stranded RNA, respectively. After 5 min of incubation, DNA fluorescence of the sample was analysed using a Partec CyFlow Space flow cytometer (532 nm green solid-state laser, operating at 30 mW; Partec GmbH, Görlitz, Germany). Using Partec FloMax software v2.4d (Partec GmbH, Münster, Germany) the following four histograms were obtained: fluorescence pulse integral in linear scale (FL); forward light scatter (FS) vs. side light scatter (SS), both in logarithmic (log) scale; FL vs. time; and FL vs. SS in log scale **[see** **Supporting Information—Fig. S1****]**. To digitally remove some of the debris, the FL histogram was gated using a polygonal region defined in the FL vs. SS histogram (see R1 in **Supporting Information—Fig. S1**), and was further applied to all the other graphics. At least 1300 nuclei in both sample and standard G_1_ peaks were analysed per sample (Suda *et al.* 2007). Only coefficient of variation (CV) values of G_1_ peak of *G. communis* below 5 % were considered acceptable (see examples in **Supporting Information—Fig. S1**), otherwise a new sample was prepared and analysed until this quality standard was achieved (Greilhuber *et al.* 2007).

Genome size was estimated in 41 populations by analysing three plants per population and cytotype individually (rarely less, unless there were no more plants in the locality, while in a few populations up to 30 individuals were analysed for genome size) **[see** **Supporting Information—Table S2****]**. For the remaining individuals and populations, only DNA ploidy level information was gathered following the pooled sample strategy (5–6 individuals plus the reference standard). A total of 108 natural populations of *G. communis* and 2665 individuals were sampled and analysed **[see** **Supporting Information—Table S1****]**.

We used flow cytometry to measure DNA ploidy of offspring produced by plants of known ploidy. A total of 1252 seeds from 178 individuals from four tetraploid, two hexaploid and four octoploid pure-ploidy populations and one tetraploid–octoploid mixed population were analysed. We sampled 10–15 seeds per maternal individual, and 7–15 individuals per population and cytotype. For pure-ploidy populations of tetraploids and octoploids and mixed-ploidy population, five seeds were chopped simultaneously with the internal reference standard (pooled sample strategy) following the protocol described above, producing easy to interpret histograms. When analysing the seeds, at least two peaks (plus the peak of the internal standard) were always obtained, corresponding to the peak of the embryo and that of the endosperm. Consequently, the interpretation of each histogram was made with particular caution, determining the ploidy levels of all the peaks obtained in the histogram. Preliminary analyses revealed that hexaploid populations presented higher variability and thus only two seeds were pooled, in order to unambiguously assign the DNA ploidy levels of each seed.

The holoploid genome size (2C in pg; *sensu*Greilhuber *et al.* 2005) was obtained using the following formula:

$$\text{ Holoploid genome size (pg)}\;=\;\frac{\text{G}{\text{. communis G}}_{\text{1}}\text{ peak mean}}{{\text{reference standard G}}_{\text{1}}\text{ peak mean}}\times \;\text{reference standard genome size}$$

Based on the chromosome counts obtained in this study and respective genome sizes, as well as the four chromosome numbers described in the literature for *G. communis* and *G. italicus*, DNA ploidy levels were inferred for each sample and individual. Populations were then characterized according to their DNA ploidy composition.

Descriptive statistics of holoploid genome size were calculated for each cytotype and species (mean, standard deviation of the mean, coefficient of variation of the mean, maximum and minimum values) based on the individual flow cytometric estimates. Mean and standard deviation of the mean were also calculated for the monoploid genome size (1C*x*; holoploid genome size divided by inferred DNA ploidy level; *sensu*Greilhuber *et al.* 2005). Differences in holoploid and monoploid genome sizes among species and cytotypes were investigated using linear models (hereafter LM) performed in R software version 3.0.1 (R Core Development Team 2016), using the packages ‘car’ for Type-III analysis of variance (Fox *et al.* 2015), ‘lme4’ for generalized linear models (GLMs; Bates *et al.* 2014) and ‘multcomp’ for multiple comparisons after Type-III analysis of variance (Hothorn *et al.* 2017).

The geographical isolation index (GI) between the two dominant cytotypes (i.e. tetraploids and octoploids) at the contact zone was calculated according to the following formula (Husband *et al.* 2016), where only pure-ploidy and mixed-ploidy populations of tetraploids and octoploids from the contact zone were considered:

$$\text{GI}\;=\;1\;-\;\frac{\text{no. mixed-ploidy populations}}{\text{total no. populations}}$$

### Environmental preferences

The environmental associations of the two dominant cytotypes were evaluated through GLM, and spatial predictive models were produced based on niche modelling tools, aiming to assess niche overlap. To explore niche overlapping, two approaches were used considering two different spatial scales: (i) one with an extension encompassing the contact zone in Central Portugal; and (ii) the other extension encompassing the entire territory of mainland Portugal.

Variables were extracted from the following sources with a resolution of ~111 m: (i) bioclimatological data from http://home.isa.utl.pt/~tmh/aboutme/Informacao_bioclimatologica.html (methodology to obtain variables in Monteiro-Henriques *et al.* 2016); and (ii) data for soil conditions from: http://epic-web gis-portugal.isa.ulisboa.pt/. Values for climatic and soil variables were extracted for all the surveyed populations using the R package ‘dismo’ (Hijmans *et al.* 2017). Then, GLMs were used to explore climatic and soil variables and assess differences between tetraploid and octoploid populations (Table 1), namely for climatic variables [mean annual total precipitation (PP), mean temperature of the hottest month of the year (Tmax), mean temperature of the coldest month of the year (Tmin), mean maximum temperature of the coldest month of the year (M), mean minimum temperature of the coldest month of the year (m)], bioclimatic indexes [continentality index (IC), compensated thermicity index (ITC), summer ombrothermic index (Ios3)], soil conditions [texture (txt) and pH] and altitude. Correlation between variables was explored using Pearson coefficient for continuous variables and Spearman’s ρ for categorical variables, to assist variable selection by removing variables with correlation values higher than 0.7. The final set of variables selected included the following four which were also important descriptors of the type of habitat where the species grows: mean annual total precipitation, mean temperature of the hottest month, soil texture and pH (highlighted in bold in Table 1).

**Table 1.** Characterization of the climatic and soil variables for tetraploid and octoploid populations of *Gladiolus communis* in the contact zone of Central Portugal. The mean, standard error of the mean (SE) and statistical tests (comparison between cytotypes) are provided for each variable and cytotype. Significance levels: ***P < 0.01; *0.05 < *P* < 0.01; n.s., non-significant. In bold the variables used in niche modelling are highlighted. Variables were extracted from the following sources with a resolution of ~111 m: (i) bioclimatological data from http://home.isa.utl.pt/~tmh/aboutme/Informacao_bioclimatologica.html (methodology to obtain variables in Monteiro-Henriques *et al.* 2016); and (ii) data for soil conditions from: http://epic-webgis-portugal.isa.ulisboa.pt/.

Variables	Code	Tetrapoid	Octoploid	ANOVA *F*_1, 78_ value
		Mean ± SE, *n* = 43	Mean ± SE, *n* = 36	
**Precipitation**	**PP**	1096.11 ± 21.97^a^	1106.89 ± 21.74^a^	0.12 n.s.
**Mean temperature of the hottest month of the year**	**Tmax**	20.51 ± 0.13^a^	20.95 ± 0.19^b^	4.04*
Mean temperature of the coldest month of the year	Tmin	9.06 ± 0.10^a^	8.91 ± 0.12^a^	1.10 n.s.
Mean max. temp. of the coldest month of the year	M	13.52 ± 0.11 ^a^	13.42 ± 0.12^a^	0.38 n.s.
Mean min. temp. of the coldest month of the year	m	4.61 ± 0.08^a^	4.50 ± 0.09^a^	0.84 n.s.
Continentality index	IC	11.44 ± 0.13^a^	12.04 ± 0.19^b^	6.95*
Compensated thermicity index	ITC	327.12 ± 2.78^a^	325.81 ± 3.22^a^	0.10 n.s.
Summer ombrothermic index	Ios3	1.10 ± 0.03^a^	1.09 ± 0.03^a^	0.05 n.s.
**Soil texture**	**Texture**	2.14 ± 0.29^a^	2.25 ± 0.13^a^	0.11 n.s.
**Soil pH**	**pH**	308.60 ± 46.51^a^	99.44 ± 19.69^b^	15.02***
Altitude	Alt	198.61 ± 18.63^a^	169.23 ± 17.38^a^	0.94 n.s.
Latitude	Lat	−8.47 ± 0.05^a^	−8.58 ± 0.03^a^	3.14 n.s.
Longitude	Long	39.98 ± 0.05^a^	40.01 ± 0.04^a^	0.18 n.s.

Spatial predictive models were calibrated based on presence/absence records collected in the field and the selected environmental and soil variables (Table 1). For the tetraploid data set, tetraploid populations were recorded as presences and octoploid populations as absences, and vice versa for the octoploid data set. Mixed tetraploid–octoploid populations were considered as presences for both cytotypes. For the contact zone (Central Portugal) we used data from 76 sampling points (including 33 tetraploid, 40 octoploid and 3 tetraploid–octoploid populations), corresponding to all the known occurrences of *G. communis* with a minimum distance between populations of 600 m. For the territory of Portugal, and aiming to reduce the bias effect of spatial clustering associated with our intense screening in the contact zone, only occurrences that had a minimum distance of 10 km between them were selected, based on radon selection, resulting in a subset of 66 sampling points (including 35 tetraploid, 19 octoploid and 6 tetraploid–octoploid populations).

Environmental niche modelling (ENM) of tetraploids and octoploids was created using R package ‘biomod2’ (Thuiller *et al.* 2016). Final model for each cytotype is based on the combination of results from different modelling techniques, each one replicated 30 times after data splitting into training (70 %) and testing (30 %) subsets based on random selection, aiming to reduce uncertainty and to produce robust models (Phillips *et al.* 2006; Araújo and New 2007). In the resampling replication, each specific occurrence was used only once in each run, as training or as test without replacement, making all replicates statistically independent (Phillips 2008). Models were evaluated based on the independent accuracy measure AUC of ROC (area under the curve of the receiver operating characteristic), and only those with AUC > 0.7 were used in the ensemble forecasting procedure, the approach used to produce the final model for each cytotype.

Model evaluation revealed high ROC values (contact zone: 4*x*—0.79 ± 0.01 and 8*x*—0.79 ± 0.01; Portugal: 4*x*—0.77 ± 0.01 and 8*x*—0.76 ± 0.01) and relatively low omission rates (contact zone: 4*x*—0.19 ± 0.02 and 8*x*—0.28 ± 0.02; Portugal: 4*x*—0.23 ± 0.01 and 8*x*—0.28 ± 0.01). However, when considering the binary projections, the omission rates decrease to 0.10 and 0.09 for the tetraploid and octoploid models in the contact zone, respectively, and 0.17 and 0.04 in Portugal (tetraploids and octoploids, respectively), demonstrating that the models were able to predict the occurrences with high accuracy, namely for octoploids. The binary projection produced by the final model of each cytotype was used to calculate niche overlap.

Cytotype niche overlap was quantified through the metric of proportional similarity of the distribution of both cytotypes, using Schoener’s *D* (a measure of niche similarity; Schoener 1970). This metric ranges from zero (no overlap) to one (complete overlap). The ‘ecospat’ (Broennimann *et al.* 2012) and ‘raster’ (Hijmans et al. 2017) packages were used to perform niche identity and similarity tests (Warren *et al.* 2008; Broennimann *et al.* 2012). In niche equivalency (identity test), the points of both cytotypes were pooled and randomly split in two groups according to size of the original data set. This new data set was used in *D* calculation, and the process was repeated 100 times (to obtain confidence intervals that enable evaluation of the null hypothesis). The resulting *D* values (simulated values) were compared with the observed *D* value, and cytotype niches were considered equivalent if the observed *D* value fell within the 95th percentile of the simulated *D* values (Broennimann *et al.* 2012). In niche similarity (similarity test), we evaluate if the environmental niches of the two cytotypes were distinguishable from each other. In this case, the comparison was between the points of one cytotype and random points from the geographic range of the other cytotype. As in the identity test, the process was repeated 100 times and *D* values were calculated. The results revealed if niche overlap between the cytotypes is greater (niche conservation) or lower (niche divergence) than expected, according to the geographic region of the other cytotype. All the models and analyses were performed in R software version 3.0.1 (R Core Development Team 2016).

### Reproductive success in natural populations

The reproductive success of each cytotype was evaluated in 11 natural populations, namely 10 pure-ploidy populations (including four tetraploid, two hexaploid and four octoploid populations) and one mixed-ploidy population composed by tetraploid and octoploid individuals (MC201). In each population, 11–20 individuals of known ploidy level were labelled and infructescences collected in individually labelled bags. The number of fruits was counted for each inflorescence and fruit set calculated as the proportion of flowers that developed into fruit. The number of morphologically viable seeds (based on their size and shape) was assessed in all fruits, and the seed:ovule ratio (S:O ratio) was calculated by dividing the number of morphologically viable seeds by the number of ovules. The total reproductive success of populations and cytotypes was also calculated by multiplying the S:O ratio by the fruit set. Descriptive statistics were calculated for each population type.

Differences in fruit set, S:O ratio and total reproductive success between the three cytotypes (tetraploids, hexaploids and octoploids) within pure-ploidy populations, differences between tetraploids and octoploids in the mixed-ploidy population, and differences between pure- and mixed-ploidy populations (excluding hexaploid ones) were assessed using GLM. Mixed models with individual and population as random factors were initially used, but the random factors were further removed due to low variance in comparison with residuals (Bolker *et al.* 2009). A binomial distribution with a logit link function was used for fruit set, and a Gaussian distribution with an identity link function was used for S:O ratio and total reproductive success after transformation with the arcsine of the square root. When significant differences were obtained, *post hoc* tests for multiple comparisons were performed.

All analyses were performed in R software version 3.0.1 (R Core Development Team 2016), using the packages ‘car’ for Type-III analysis of variance (Fox and Weisberg 2015), ‘lme4’ for GLMs (Bates *et al.* 2014) and ‘multcomp’ for multiple comparisons after Type-III analysis of variance (Hothorn *et al.* 2017).

## Results

### Genome size and cytogenetic diversity

**Figure 1.**
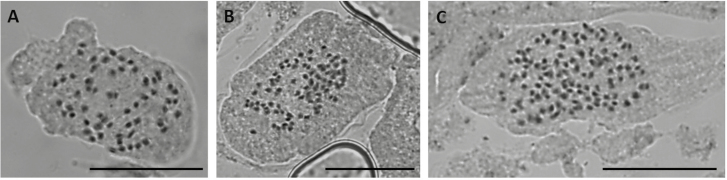
*Gladiolus communis* chromosome counts. (A) tetraploid (2*n* = 4*x* = 60 chromosomes), (B) hexaploid (2*n* = 6*x* = 90) and (C) octoploid (2*n* = 8*x* = 120) individuals. Bar = 20 µm.

**Figure 2.**
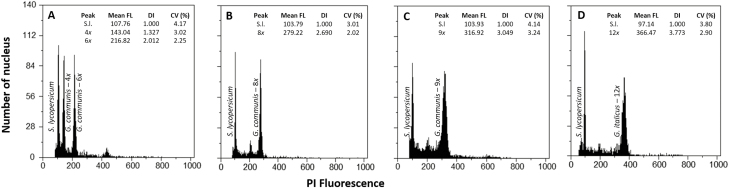
Flow cytometric histograms of relative propidium iodide fluorescence from nuclei isolated from fresh leaves of *Solanum lycopersicum* ‘Stupické’ (S.l.) and different cytotypes and/or species of *Gladiolus*: (A) tetraploid (4*x*) and hexaploid (6*x*), (B) octoploid (8*x*) and (C) nonaploid (9*x*) individuals of *G. communis*, and (D) duodecaploid (12*x*) individual of *G. italicus*.

Based on chromosome counts and flow cytometric analyses, we detected three ploidy levels in *G. communis*: tetraploids with 2*n* = 4*x* = 60 chromosomes (Fig. 1A) and an average genome size of 2.69 ± 0.06 pg/2C (mean ± SD); hexaploids with 2*n* = 6*x* = 90 chromosomes (Fig. 1B) and an average genome size of 4.07 ± 0.07 pg/2C; and octoploids with 2*n* = 8*x* = 120 chromosomes (Fig. 1C) and an average genome size of 5.42 ± 0.14 pg/2C (Table 2; Fig. 2A and B) **[see** **Supporting Information—Table S2****]**. Genome size estimates also suggest the occurrence of nonaploid *G. communis* individuals, characterized by genome sizes with nine times the monoploid genome size (1C*x*) values obtained for the other ploidy levels, and had mean genome size of 6.10 ± 0.18 pg/2C (Table 2; Fig. 2C). These individuals were rare and we were unable to confirm their ploidy using chromosome counts. *Gladiolus italicus* had a higher genome size (2C = 7.27 ± 0.17 pg) than *G. communis*, consistent with duodecaploids, as described for the species (Table 2; Fig. 2D). The holoploid genome sizes (2C) of the five cytotypes differed significantly (*F*_4, 175_ = 7691.3, *P* < 0.001; Table 2). Monoploid genome size values were conserved within *G. communis*, with no significant differences being observed between cytotypes (*F*_3, 155_ = 7691.3, *P* = 0.5272; Table 2). However, monoploid genome size of *G. communis* (0.67 ± 0.03 pg) was significantly higher than for *G. italicus* (0.61 ± 0.01 pg; *F*_1, 178_ = 7691.3, *P* < 0.001).

**Table 2.** Genome size and DNA ploidy level estimations for *Gladiolus communis* and *G. italicus*. Holoploid genome size (G.s.; 2C) is provided for each species and cytotype as mean and standard deviation (SD) in picograms (pg), followed by coefficient of variation (CV, %), DNA range (minimum, Min, and maximum, Max, genome size values); mean and standard deviation of the mean is also provided for the monoploid genome size (1C*x*); total number of individuals (N total) and populations (N pop) analysed for genome size are also provided for each ploidy level. Chromosome numbers (Chr. number) for each species based on chromosome counts of this study and bibliographic records are also provided. DNA ploidy levels: tetraploid (4*x*), hexaploid (6*x*), octoploid (8*x*), nonaploid (9*x*) and duodecaploid (12*x*). Different letters denote statistically significant differences at *P* < 0.05. ^1^Chromosome numbers detected in this study; ^2^Chromosome counts documented in the bibliography; ^3^DNA ploidy level extrapolated based on the genome size values obtained here and on the chromosome counts available from other ploidy levels.

Species	DNA ploidy level	Chr. number	Holoploid G.s. (2C, pg)					Monoploid G.s. (1C*x*, pg)		N total	N pop
			Mean	SD	CV (%)	Min	Max	Mean	SD		
*G. communis*	4*x*	60^1,2^	2.69^a^	0.06	2.28	2.58	2.86	0.67^a^	0.02	57	16
*G. communis*	6*x*	90^1,2^	4.07^b^	0.07	1.76	3.93	4.19	0.68^a^	0.01	9	3
*G. communis*	8*x*	120^1,2^	5.42 ^c^	0.14	2.53	5.13	5.73	0.68^a^	0.02	91	21
*G. communis*	9*x*	135^3^	6.10^d^	0.18	2.89	5.98	6.23	0.68^a^	0.02	2	1
*G. italicus*	12*x*	180^2^	7.27^e^	0.17	2.31	6.97	7.55	0.61^b^	0.01	21	10

### Geographic distribution of cytotypes

**Figure 3.**
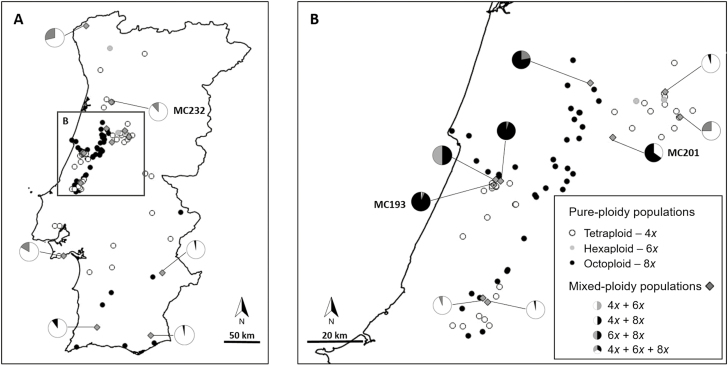
*Gladiolus communis* cytotype screening: (A) all studied area (Portugal); and (B) detail of the contact zone studied (Central Portugal). White, grey and black circles represent pure tetraploid, hexaploid and octoploid populations, respectively. Mixed-ploidy populations are represented by a grey diamond and each population is accompanied by a pie diagram reflecting cytotype composition. One sole population harbouring also two nonaploid individuals (not included in the pie diagram) is denoted by a dotted grey diamond, namely population MC193. Populations identified with ID code correspond to the populations where all the individual plants were sampled in detail (see Fig. 4). DNA ploidy levels: tetraploid (4*x*), hexaploid (6*x*), octoploid (8*x*).

Tetraploids and octoploids were prevalent across the geographic area sampled, both occurring in pure- and in mixed-ploidy populations (Fig. 3). No marked segregation pattern of cytotype arrangement in space was observed: tetraploids seem to occur across the entire area surveyed, and octoploids in the centre and south of the surveyed area, forming broad contact zones. Minority cytotypes were also detected, namely hexaploids, which were observed growing with other cytotypes and occasionally found forming pure populations (Fig. 3). A few nonaploids in a mixed-ploidy population harbouring all cytotypes of *G. communis* were also detected (Fig. 3) **[see** **Supporting Information—Table S3****]**. Most populations were cytogenetically uniform (i.e. pure-ploidy populations, 86.1 %) and, in the majority of cases, were composed of either tetraploid or octoploid individuals (43.5 and 39.8 %, respectively). Hexaploids were detected growing alone in three locations (2.8 %) (Fig. 3). Populations harbouring two or more cytotypes (i.e. mixed-ploidy populations) represented 13.9 % of all sampled populations. The mixed-ploidy populations presented different cytotype compositions: tetraploids and hexaploids (4.6 %), in which the former is more frequent than the latter; tetraploids and octoploids (5.6 %) again, in which tetraploids are generally more abundant than octoploids, except in one population; tetraploids, hexaploids, octoploids and nonaploids (0.9 %; one population), where octoploids are the dominant cytotype; and hexaploids and octoploids (2.8 %), in which octoploids are dominant, except in one location where only two plants, one of each cytotype, were found **[see** **Supporting Information—Table S3****]**.

Within the contact area (Fig. 3B), most localities contained a single ploidy of either tetraploids (42.0 %), octoploids (44.4 %) or rarely hexaploids (2.5 %). These populations were distributed mostly in parapatry; still, cytotypes were found growing in sympatry in some locations (11.1 %) (Fig. 3B). Octoploid populations occur from north to south, resulting in cytogenetically diverse contact zones with tetraploids to the east, south and south-west. At these contact zones, areas with different types of mixed-ploidy populations were detected. Hexaploids were frequent in the contact zones between tetraploids and octoploids, although they were also detected in other places of the screened area, growing with tetraploid individuals. Tetraploids and octoploids, the two main cytotypes, were observed growing together in four locations out of the 81 populations at the contact zone (4.9 %), resulting in a total GI of 0.95, with tetraploids and octoploids presenting a similar individual geographical isolation index (GI_4*x*_ = 0.90, GI_8*x*_ = 0.91).

**Figure 4.**
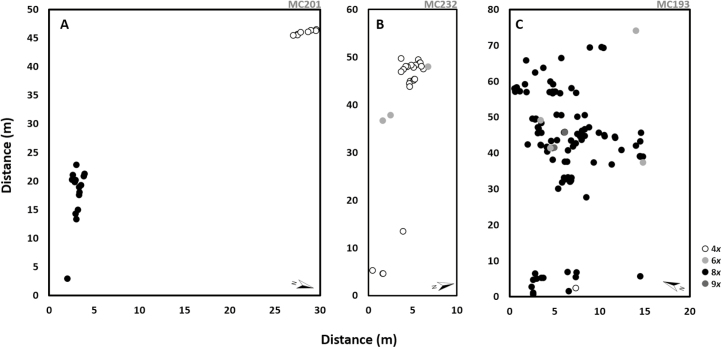
Fine-scale distribution of *Gladiolus communis* individuals within three mixed-ploidy populations: (A) tetraploid and octoploid mixed-ploidy population (MC201), (B) tetraploid and hexaploid mixed-ploidy population (MC232); and (C) tetraploid, hexaploid, octoploid and nonaploid mixed-ploidy population (MC193). Each point represents one individual plant mapped in a *x/y* system where distance is given in metres (m): tetraploids (4*x*), hexaploids (6*x*), octoploids (8*x*) and nonaploids (9*x*) individuals are represent by white, grey, black and dark grey points, respectively.

The detailed screening of three selected mixed-ploidy populations revealed variable patterns of cytotype distribution within each population (Fig. 4). In the tetraploid–octoploid population (MC201), cytotypes were distributed in two well-defined clusters separated by >20 m, with tetraploids being restricted to the north-east side and octoploids to the south-west of the population (Fig. 4A). The mixed-ploidy population with tetraploids and hexaploids (MC232) was dominated by tetraploid individuals, with a few hexaploids growing intermingled (Fig. 4B). The population with the highest cytogenetic diversity (MC193) revealed to be dominated by octoploid individuals with a few tetraploid, hexaploid and nonaploid plants growing intermingled (Fig. 4C). While MC201 and MC193 were located in the contact zones, MC232 is located in an otherwise tetraploid zone (Fig. 3).

### Environmental preferences

**Figure 5.**
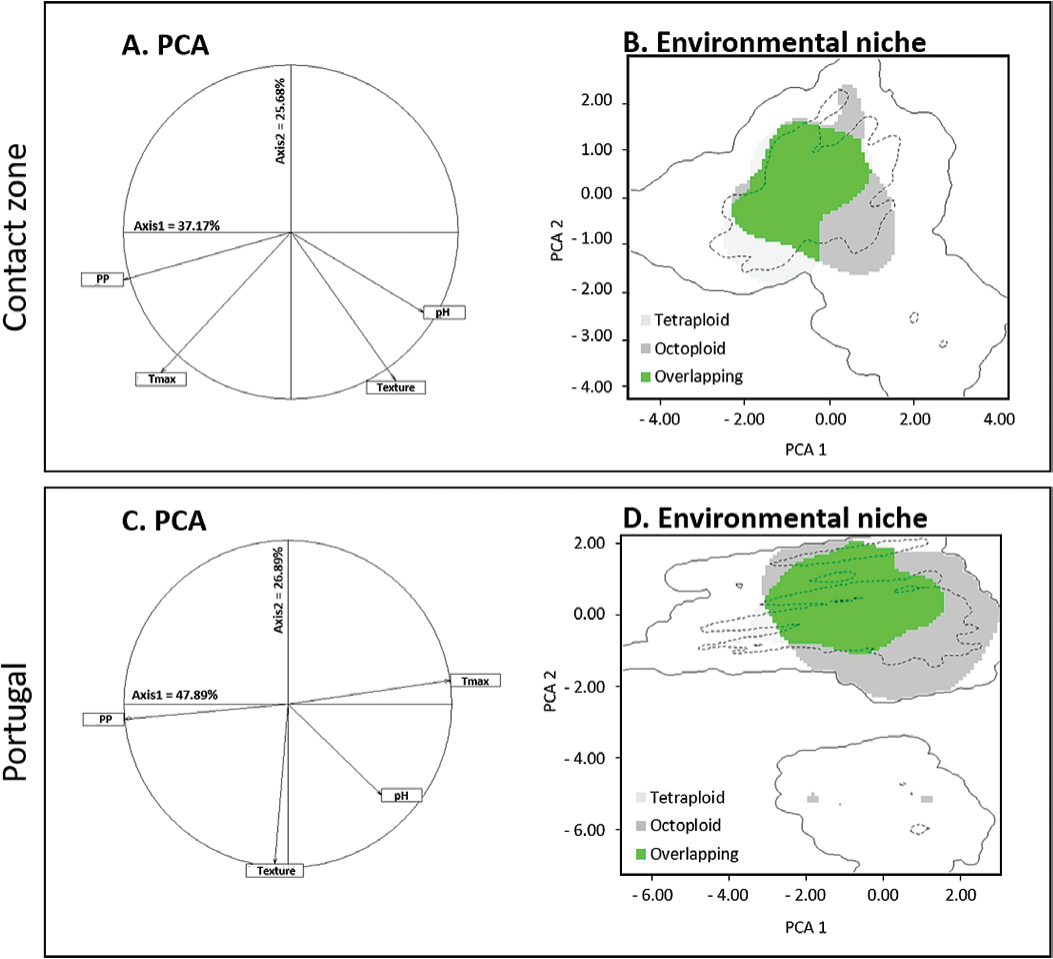
Results of ecological niche models for *Gladiolus communis* polyploid complex at (A, B) the contact zone in Central Portugal, and (C, D) Portugal. (A) and (C) represent the contribution of climatic and soil variables in the first two axes of the principal component analyses (PCA) and the percentage of variance explained by each axis. (B) and (C) represent the environmental niche of each cytotypes based on the PCA of selected variables; coloured areas represent suitable habitats as follows: light grey—tetraploids; dark grey—octoploids; and green—overlapping areas between tetraploids and octoploids; the continuous line corresponds to the whole climatic space, while the dashed line indicates the 75th percentile.

Niche geographic overlap between tetraploids and octoploids at both the contact zone (Schoener’s *D* metric, *D* = 0.03) and Portugal (*D* = 0.01) was low (Table 3). However, and despite little geographical overlap, there was no statistical evidence that the environmental niches differed, i.e. neither niche equivalency nor niche similarity was rejected (Table 3). This indicates that environmental niche of the dominant cytotypes was equivalent within the suitable ranges of both tetraploids and octoploids, and that environmental niche of each cytotype was similar to the suitable range of the other cytotype. At the contact zone, the selected climatic and soil variables explained 62.98 % of the variance in the distribution (Fig. 5A), and a high environmental overlap of a given cytotype within the niche of the opposite cytotype was observed (74.87 and 61.95 % for tetraploids and octoploids, respectively; Fig. 5B). A similar pattern was observed for Portugal, although the climatic and soil variables explained higher variance than at the contact zone (74.78 %; Fig. 5C). A high environmental overlap between cytotypes was also observed (91.51 and 47.96 % for tetraploids and octoploids, respectively; Fig. 5D).

**Table 3.** Niche analyses in *Gladiolus communis*. For each region studied, equivalency (*D* and *P* values) and similarity (*P* value) tests for suitable habitat are given.

Suitable habitat	Equivalence test		Similarity test (*P* values)	
	*D* value	*P* value	Tetra -> Octo	Octo -> Tetra
Contact zone	0.034	0.960	0.406	0.337
Portugal	0.009	0.515	0.535	0.515

### Reproductive success in natural populations and offspring cytogenetic composition

**Figure 6.**
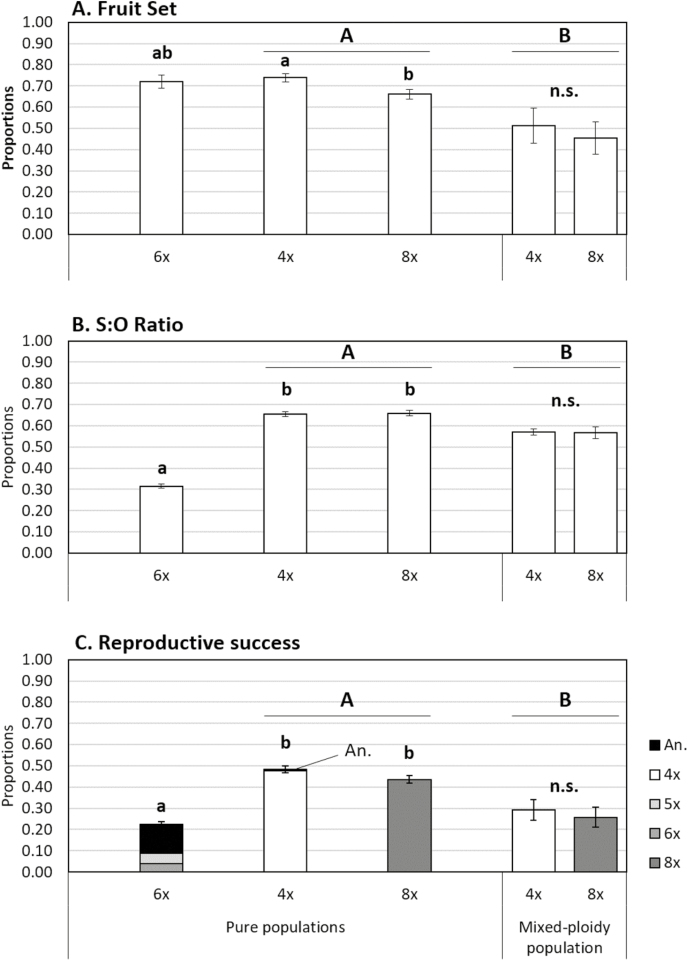
Reproductive fitness of natural pure- and mixed-ploidy populations of *Gladiolus communis*: (A) fruit set; (B) S:O ratio (number of viable seeds divided by the number of ovules); and (C) reproductive success (fruit set multiplied by S:O ratio). In (C) the proportion of DNA ploidy levels detected in the offspring is also given. DNA ploidy levels: tetraploid (4*x*), pentaploid (5*x*), hexaploid (6*x*), octoploid (8*x*); seeds with genome size values out of the range of variation of each ploidy levels were assumed as aneuploids (An.). Different letters correspond to statistically significant differences as follows: (i) differences between population type (pure- vs. mixed-ploidy populations, excluding 6*x*) are denoted by upper case letters; and (ii) differences between ploidy levels within population type (among 4*x*, 6*x* and 8*x* from pure populations, and between 4*x* and 8*x* from the mixed-ploidy population) are denoted by lower case letters (Tukey HSD; *P <* 0.05); n.s. correspond to non-significant differences (*P* > 0.05).

Plants in all the natural populations successfully formed fruits and seeds. However, the success differed according to the cytotype and population type. Pure-ploidy populations (excluding the hexaploid populations) had higher reproductive success compared to the mixed-ploidy population for all parameters (fruit set: *F*_1, 1033_ = 15.51, *P* < 0.001; S:O ratio: *F*_1, 706_ = 4.62, *P* = 0.032; reproductive success: *F*_1, 1033_ = 21.04, *P* < 0.001; Fig. 6). Within pure-ploidy populations, significant differences between cytotypes were observed for all the variables (fruit set: *F*_2, 1087_ = 4.96, *P* = 0.007; S:O ratio: *F*_2, 770_ = 100.18, *P* < 0.001; reproductive success: *F*_2, 1087_ = 28.34, *P* < 0.001), with octoploids having lower fruit set than tetraploids. S:O ratio and reproductive success were similar in tetraploids and octoploids (*P >* 0.05), but significantly higher than for hexaploids (*P <* 0.05; Fig. 6B and C). Within the mixed-ploidy population, no significant differences were observed between the cytotypes for any of the reproductive variables (fruit set: *F*_1, 79_ = 0.27, *P* = 0.603; S:O ratio: *F*_1, 37_ = 0.01, *P* = 0.934; reproductive success: *F*_1, 79_ = 0.15, *P* = 0.698).

The analyses of offspring ploidy revealed that tetraploid and octoploid individuals, in both pure-ploidy and mixed-ploidy populations, produced seeds with the same ploidy as the mother plants (Fig. 6C). Tetraploid plants in pure populations produced a few aneuploids (<1 % of the offspring; Fig. 6C) **[see** **Supporting Information—Table S4****]**. In contrast, the flow cytometric analyses of the seeds from hexaploid individuals pointed out highly variable genome sizes, the analyses of the genome size estimates suggest the following DNA ploidy levels: 62 % of seeds were aneuploid, 20 % were pentaploids and 18 % were hexaploid, although further confirmation is needed.

## Discussion

This study corroborates the existence of high cytogenetic diversity within the *G. communis* polyploid complex. Two dominant cytotypes, tetraploids and octoploids, were observed along with two minority cytotypes, mostly hexaploids, and rarely nonaploids. Tetraploids and octoploids have been well documented on the Iberian Peninsula through chromosome counts (Fernandes *et al.* 1948; Fernandes 1950; Fernandes and Queirós 1971; Nilsson and Lassen 1971; Löve and Kjellqvist 1973; Queirós 1980; Fernández *et al.* 1985). Also, hexaploids have been previously reported in the Mediterranean basin (Darlington and Wylie 1955). We observed them in 11 % of the sampled localities (12 of 105 localities), commonly growing with one of the dominant cytotypes and occasionally in pure-ploidy populations. Nonaploids are reported here for the first time and were detected in the most diverse mixed-ploidy population.

Despite the cytogenetic diversity reported in *G. communis*, almost nothing was known about the geographic distribution of the cytotypes, or the presence and structure of its contact zones. Based on our survey, tetraploids occurred throughout the sampling area, although they were more common in the north and central regions of Portugal. Octoploids occurred in south and central regions of Portugal, but not in the north, notwithstanding the fact that more extensive surveys are needed to confirm this pattern. Although several mixed-ploidy populations were found, the geographical isolation index between tetraploids and octoploids is high, reflecting the fact that most of the populations contain a single cytotype. These populations distribute in space allopatrically or parapatrically, forming several contact zones between tetraploids and octoploids. However, despite that tetraploids and octoploids have non-overlapping distributions, they can inhabit similar environmental niches. Niche identity and similarity tests showed that tetraploids and octoploids occupy similar niches and are not differentiated in their environmental niches, showing niche conservation. These results contrast with other polyploid complexes for which niche differentiation, driven either by the direct effects of polyploidy or by subsequent selection, underlies the spatial separation of cytotypes and allows them to escape the minority cytotype disadvantage (e.g. Glennon *et al.* 2012; Thompson *et al.* 2014; Visger *et al.* 2016; Muñoz-Pajares *et al.* 2017). Still, the absence of environmental niche differences might not be completely unexpected as polyploids might not differ from their lower ploidy ancestors, either because they have been formed recently and the new polyploids did not have time to diverge from their progenitors, because genome duplications did not generate significant direct physiological changes, and/or because they might have been subjected to recurrent gene flow (Godsoe *et al.* 2013; Laport *et al.* 2016). Also, the effect of other environmental parameters on the distribution patterns observed in *G. communis* cannot be completely ruled out, nor the fact that niche differentiation might occur at a special resolution higher than that used in our study, although we did not find any clear evidence of differentiation in the field, namely considering the type of vegetation or the type of substrate in the mixed-ploidy populations detected (M. Castro, field observations).

Considering that *G. communis* cytotypes do not differ in suitable habitat, there should be historical processes and other ecological determinants shaping their distributional patterns, similarly to what has been observed in several polyploid complexes (e.g. Baack 2004, 2005; Pannell *et al.* 2004; Baack and Stanton 2005; Godsoe *et al.* 2013; Münzbergová *et al.* 2013; Wefferling *et al.* 2017). Contact zones are generated by direct emergence of neopolyploids in lower ploidy parental populations or through secondary contact of previously allopatric distributions in which cytotypes colonized the area separately in dissimilar ways and at different timings (Petit *et al.* 1999; Lexer and van Loo 2006). Although we still do not know the origin of *G. communis* contact zones, the different cytotype compositions found in natural populations provide significant insights into the processes that might be occurring at these areas (e.g. Husband and Schemske 1998; reviewed in Husband *et al.* 2013; Suda *et al.* 2013). One of the main observations is the fairly few mixed-ploidy populations (10 vs. 90 % of mixed- and pure-ploidy populations), all composed of unbalanced number of tetraploid and octoploid plants (either dominated by tetraploid or by octoploids). In the absence of environmental differences, and regardless of the origin of the contact zone, *G. communis* mixed tetraploid–octoploid populations are expected to be more common at contact areas than detected here (4.9 % in the contact zone and 6.5 % from the total), since cytotypes might disperse to areas of the other cytotype and/or new cytotypes might be formed. Consequently, the high geographical isolation observed between *G. communis* cytotypes suggests that the mixed-ploidy populations might be transitory because strong frequency-dependent selection is expected to eliminate the minority cytotype as a result of fitness disadvantage generated by its lower number. This selection will ultimately drive the occurrence of pure-ploidy populations at contact zones (Levin 1975; Husband 2000).

However, tetraploid–octoploid populations may persist in nature. The regular production of unreduced gametes and the presence of reproductive barriers promoting assortative mating might lessen the magnitude of frequency-dependent selection and enable cytotype coexistence (e.g. Felber 1991; Segraves and Thompson 1999; Husband 2004; Husband and Sabara 2004; Kennedy *et al.* 2006). Octoploids might emerge directly in tetraploid populations through the union of two unreduced gametes (*n* = 4*x*) or might result from seed dispersal from neighbouring octoploid populations. Unreduced gamete production has been detected in controlled pollinations in tetraploid *G. communis* (M. Castro *et al.*, in preparation) and in screenings in natural populations through the detection of hexaploid individuals (see below). The rates at which unreduced gametes are produced might feed the population of octoploids enabling their maintenance within tetraploid populations (Felber 1991; Husband 2004). Additionally, seed ploidy analyses in a tetraploid–octoploid population suggest that strong reproductive barriers may enforce assortative mating, further favouring cytotype coexistence. Reproductive barriers driven, for example, by phenological and/or morphological mismatch, different pollinator assemblages or preferences, and/or gametic isolation will, thus, play a major role for overcoming minority cytotype exclusion in mixed-ploidy populations. Therefore, the fate of octoploids might depend not only on the rates of unreduced gamete formation but also on the reproductive isolation levels. Additionally, differences in other traits, such as perenniality or asexual reproduction, could compensate for the minority cytotype disadvantage (e.g. Rodriguez 1996; Kao 2007; Castro *et al.* 2016a). In other polyploid complexes, traits such as the production of bulbs represented an advantage, enabling new cytotypes to persist at initial stages and spread within lower ploidy populations (e.g. *Allium oleraceum*; Duchoslav *et al.* 2010; *G. × sulistrovicus*; Szczepaniak *et al.* 2016). If, through some of these traits, the number of octoploids can surpass the number of tetraploids, at some time octoploids might even outcompete tetraploids and exclude them from the population, as observed in other polyploid complexes (e.g. Buggs and Pannell 2007). Indeed, octoploids were observed as the dominant cytotype in some mixed-ploidy populations of the contact zone. Future studies on the contribution of all the above-mentioned processes, and on the relative contribution of sexual vs. asexual reproduction for the maintenance of the populations, are needed to fully understand the dynamics of mixed-ploidy populations.

The cytotype composition of *G. communis* natural populations also revealed that hexaploid plants might be more common than previously thought. These hexaploids might have originated through two different pathways. Hexaploids may originate from tetraploids through the union of reduced (*n* = 2*x*) and unreduced (*n* = 4*x*) gametes (Ramsey and Schemske 1998). Indeed, unreduced gamete formation is an important pathway for new polyploid emergence and has been shown to be common in nature (Felber 1991; Bretagnolle and Thompson 1995; Ramsey and Schemske 1998; Husband 2004; Ramsey 2007). This might explain the detection of hexaploid plants frequently found in otherwise tetraploid populations. Alternatively, hexaploids may form as a result of hybridization events between tetraploid and octoploid *G. communis* individuals. *Gladiolus communis* is pollinated by generalist pollinators that seem to have no cytotype preferences and might move pollen within mixed-ploidy populations or between populations in close proximity (M. Castro *et al.*, in preparation). Additionally, controlled pollinations between tetraploid and octoploid plants were also successful in producing hexaploid offspring (M. Castro *et al.*, in preparation). Either one of these pathways, i.e. unreduced gamete formation or hybridization, may operate in natural populations, being difficult to distinguish them without genetic markers. However, the relative abundance of tetraploid–hexaploid populations and paucity of tetraploid–hexaploid–octoploid populations suggests that the majority of the hexaploids are formed through unreduced gametes in tetraploid populations. Additionally, unreduced gamete production has been frequently detected in controlled pollinations involving tetraploid *G. communis* (M. Castro *et al.*, in preparation), supporting it as a probable pathway for new cytotype emergence in natural populations. Quantifying unreduced gamete production in natural populations will provide significant insights on how frequent this process could be involved with hexaploid emergence.

Interestingly, hexaploid individuals were also found forming pure-ploidy populations, showing that this cytotype can successfully establish and spread beyond parental populations, although their sexual reproductive fitness was revealed to be lower in comparison with tetraploids and octoploids. Regardless of the lower fitness, recurrent unreduced gamete formation and asexual reproduction might enable to compensate for this disadvantage (e.g. Husband 2004; Kao 2007; Castro *et al.* 2016a). The successful establishment of hexaploid plants further contributes to the diversification of the complex. Ultimately, contact zones result from the combination of several factors, including historical factors, unreduced gamete formation, pollen flow and hybridization events, and seed dispersal, among others (Petit *et al.* 1999; Levin 2002; Lexer and van Loo 2006). Future studies reconstructing the history of the complex and quantifying unreduced gamete production, and its ability to hybridize, would provide significant insights on the dynamics of the distribution of *G. communis*.

The genome size of *G. italicus* suggests that this species is duodecaploid in the studied area, which is in accordance with chromosome counts for the Iberian Peninsula (Queirós 1979; Pérez and Pastor Díaz 1994), and contrasts with the dominance of the octoploids elsewhere in the Mediterranean basin (Susnik and Lovka 1973; Strid and Franzen 1981; van Raamsdonk and de Vries 1989; Kamari *et al.* 2001). Interestingly, the variation in monoploid genome size within *G. communis* cytotypes was very low and differed significantly from that of *G. italicus* (~9 %). Given the magnitude of the differences between *G. italicus* and *G. communis*, both in ploidy levels and in monoploid genome sizes, holoploid genome size might be an important tool to detect hybridization (e.g. Kolář *et al.* 2009; Agudo-García 2017). In our study, *G. italicus* and *G. communis* were found growing in sympatry in 13 % of localities; however, all the *G. italicus* individuals were duodecaploid. In most of the cases, the duodecaploid *G. italicus* was found growing with the octoploid *G. communis* (12 out of 14 localities); still, no decaploids were observed in these localities. When growing with the tetraploid *G. communis*, no octoploid individuals with lower genome size resulting from the hybridization between the two species (~5.00 pg based on the monoploid genome sizes of each species) were observed. Although hybridization has been suggested to occur in these and in other *Gladiolus* species (e.g. van Raamsdonk and de Vries 1989; Mifsud and Hamilton 2013; Szczepaniak *et al.* 2016), we were not able to detect hybrids between *G. italicus* and *G. communis*. This suggests that, in the studied range, hybridization between them might be less common, either because of assortative mating or hybrid offspring inviability. Monoploid genome size also suggests a close relationship between the cytotypes of *G. communis*, pointing to an autopolyploid origin of the complex in the studied area. This is also supported by the high morphological resemblance between *G. communis* cytotypes (Alonso and Crespo 2010; Cantor and Tolety 2011) and by the lack of evidence supporting hybridization between *G. communis* and *G. italicus* in this region. Still, the origin of *G. communis* polyploid complex needs to be properly evaluated in future studies.

## Conclusions

In this study, we find a complex cytogeographical pattern in *G. communis*, which opens several hypotheses that might explain the formation and maintenance of its tetraploid–octoploid contact zone. According to our results, tetraploids and octoploids do not differ in their environmental requirements, potentially growing in similar habitats. Without differences in habitat requirements, mixed-ploidy populations were expected to be frequent; however, a high geographical isolation index was obtained. The high geographical isolation observed in nature, along with habitat similarity, suggests that the cytotype distribution in *G. communis* reflects historical patterns of migration and colonization, and further selection against minority cytotype, and does not result from different environmental requirements, creating a tension zone of contact. Still, in areas of contact, reproductive barriers might mediate assortative mating and enable cytotype coexistence. Nevertheless, the high cytogenetic diversity detected in the field suggests that unreduced gamete formation and hybridization events seem frequent in this complex and might be involved with recurrent polyploid formation and with gene flow between cytogenetic entities. Future studies involving reciprocal transplants will provide significant insights into the dynamics of this polyploid complex.

## Sources of Funding

This research was supported by POPH/FSE funds by the Portuguese Foundation for Science and Technology (FCT) with a doctoral grant to M.C. (SFRH/BD/89910/2012) and a starting grant and exploratory project to S.C. (IF/01267/2013), and by Project RENATURE financed by the ‘Programa Operacional Regional do Centro 2014–2020 (Centro2020) - CENTRO-01-0145-FEDER-000007’.

## Contributions by the Authors

S.C., B.H. and J.L. designed the research experiment; M.C., S.C. and J.L. developed the field and laboratory work; M.C. performed the statistical analyses; and M.C. and A.F. performed the niche modelling analyses; all the authors were involved in the discussion of the results and writing of the manuscript, approving the final document.

## Conflict of Interest

None declared.

## Supporting Information

The following additional information is available in the online version of this article—

**Figure S1.** Flow cytometry graphics analysed for each sample.

**Table S1.** Geographic information of sampled *Gladiolus* populations.

**Table S2.** Genome size variation in *Gladiolus communis.*

**Table S3.** Mixed-ploidy populations of *Gladiolus communis*.

**Table S4.** DNA ploidy levels of the offspring of pure- and mixed-ploidy populations of *Gladiolus communis*.

Supporting Information

## References

[CIT0002] Agudo-García A 2017 Evolution in anacyclus L. (Anthemideae, Asteraceae). Analysis of the contact zone between A. clavatus (Desf.) Pers. and A. valentinus L. PhD Thesis, Universidad Autonoma de Madrid, Spain.

[CIT0003] Alonso MÁ, Crespo MB 2010 *Gladiolus* L. In: Crespo MB, Herrero A, Quintanar A, eds. Flora iberica. Madrid: Real Jardín Botánico, CSIC, 185:485–491.

[CIT0004] Araújo MB, New M 2007 Ensemble forecasting of species distributions. Trends in Ecology & Evolution 22:42–47. 17011070 10.1016/j.tree.2006.09.010

[CIT0005] Baack EJ 2004 Cytotype segregation on regional and microgeographic scales in snow buttercups (*Ranunculus adoneus*: Ranunculaceae). American Journal of Botany 91:1783–1788. 21652325 10.3732/ajb.91.11.1783

[CIT0006] Baack EJ 2005 To succeed globally, disperse locally: effects of local pollen and seed dispersal on tetraploid establishment. Heredity 94:538–546. 15770232 10.1038/sj.hdy.6800656

[CIT0007] Baack EJ, Stanton ML 2005 Ecological factors influencing tetraploid speciation in snow buttercups (*Ranunculus adoneus*): niche differentiation and tetraploid establishment. Evolution 59:1936–1944. 16261731

[CIT0008] Bates D, Maechler M, Bolker B, Walker S 2014 *lme4: linear mixed-effects models using Eigen and S4* http://CRAN.R-project.org/package=lme4 (21 April 2016).

[CIT0009] Bolker BM, Brooks ME, Clark CJ, Geange SW, Poulsen JR, Stevens MH, White JS 2009 Generalized linear mixed models: a practical guide for ecology and evolution. Trends in Ecology & Evolution 24:127–135. 19185386 10.1016/j.tree.2008.10.008

[CIT0010] Bretagnolle FA, Thompson JD 1995 Gametes with the somatic chromosome number: mechanisms of their formation and role in the evolution of autopolyploid plants. New Phytologist 129:1–22. 10.1111/j.1469-8137.1995.tb03005.x 33874422

[CIT0011] Brochmann C, Brysting AK, Alsos IG, Borgen L, Grundt HH, Scheen AC, Elven R 2004 Polyploidy in arctic plants. Biological Journal of the Linnean Society 82:521–536.

[CIT0012] Broennimann O, Fitzpatrick MC, Pearman PB, Petitpierre B, Pellissier L, Yoccoz NG, Thuiller W, Fortin MJ, Randin C, Zimmermann NE, Graham CH 2012 Measuring ecological niche overlap from occurrence and spatial environmental data. Global Ecology and Biogeography 21:481–497.

[CIT0013] Buchanan A 2008 The taxonomic status of Gladiolus illyricus (Iridaceae) in Britain. Doctoral dissertation, Department of Life Sciences and the Natural History Museum, Imperial College London, London.

[CIT0014] Buggs RJ, Pannell JR 2007 Ecological differentiation and diploid superiority across a moving ploidy contact zone. Evolution 61:125–140. 17300432 10.1111/j.1558-5646.2007.00010.x

[CIT0015] Burton TL, Husband BC 2001 Fecundity and offspring ploidy in matings among diploid, triploid and tetraploid *Chamerion angustifolium* (Onagraceae): consequences for tetraploid establishment. Heredity 87:573–582. 11869348 10.1046/j.1365-2540.2001.00955.x

[CIT0105] Cantor M, Tolety J 2011 Gladiolus In: Kole C eds. Wild crop relatives: genomic and breeding resources - Plantation and ornamental crops. Berlin: Springer-Verlag Berlin Heidelberg, 133–160.

[CIT0017] Castro S, Castro M, Ferrero V, Costa J, Tavares D, Navarro L, Loureiro J 2016a Invasion fosters change: independent evolutionary shifts in reproductive traits after *Oxalis pes-caprae* L. introduction. Frontiers in Plant Science 7:874. 27446109 10.3389/fpls.2016.00874 PMC4919335

[CIT0018] Castro M, Loureiro J, Husband BC, Castro S 2016b Can we live together? The role of reproductive barriers mediating cytotype coexistence in Gladiolus communis contact zones. XII Encontro Nacional de Biologia Evolutiva, 16 December, University of Aveiro.

[CIT0019] Castro S, Loureiro J, Procházka T, Münzbergová Z 2012 Cytotype distribution at a diploid-hexaploid contact zone in *Aster amellus* (Asteraceae). Annals of Botany 110:1047–1055. 22887024 10.1093/aob/mcs177 PMC3448430

[CIT0020] Castro S, Münzbergová Z, Raabová J, Loureiro J 2011 Breeding barriers at a diploid–hexaploid contact zone in *Aster amellus*. Evolutionary Ecology 25:795–814.

[CIT0021] Darlington CD, Wylie AP 1955 Chromosome atlas of flowering plants. London, UK: George Allen and Unwin Ltd.

[CIT0022] Doležel J, Greilhuber J, Lucretti S, Meister A, Lysák MA, Nardi L, Obermayer R 1998 Plant genome size estimation by flow cytometry: inter-laboratory comparison. Annals of Botany 82(suppl_1):17–26.

[CIT0023] Doležel J, Sgorbati S, Lucretti S 1992 Comparison of three DNA fluorochromes for flow cytometric estimation of nuclear DNA content in plants. Physiologia Plantarum 85:625–631.

[CIT0024] Duchoslav M, Safárová L, Krahulec F 2010 Complex distribution patterns, ecology and coexistence of ploidy levels of *Allium oleraceum* (Alliaceae) in the Czech Republic. Annals of Botany 105:719–735. 20363760 10.1093/aob/mcq035 PMC2859911

[CIT0025] Felber F 1991 Establishment of a tetraploid cytotype in a diploid population: effect of relative fitness of the cytotypes. Journal of Evolutionary Biology 4:195–207.

[CIT0026] Fernandes A 1950 Sobre a cariologia de algumas plantas da Serra do Gerês. Agronomia Lusitana 12:551–600.

[CIT0027] Fernandes A, Garcia J, Fernandes R 1948 Herborizações nos domínios da fundação da Casa de Bragança. I-Vendas Novas. Memórias da Sociedade Broteriana 4:5–89.

[CIT0028] Fernandes A, Queirós M 1971 Sur la caryologie de quelques plantes récoltéess pendant la III^ème^ réunion de botanique péninsulare. Memórias da Sociedade Broteriana 21:343–385.

[CIT0029] Fernández I, Díez MJ, Pastor J 1985 Números cromosómicos para la flora española, 373–381. Lagascalia 13:299–302.

[CIT0030] Fox J, Weisberg S, Adler D, Bates D, Baud-Bovy G, Ellison S 2015 car: companion to applied regression http://CRAN.R-project.org/package=car. (21 April 2016).

[CIT0031] Galbraith DW, Harkins KR, Maddox JM, Ayres NM, Sharma DP, Firoozabady E 1983 Rapid flow cytometric analysis of the cell cycle in intact plant tissues. Science 220:1049–1051. 17754551 10.1126/science.220.4601.1049

[CIT0032] Glennon KL, Rissler LJ, Church SA 2012 Ecogeographic isolation: a reproductive barrier between species and between cytotypes in *Houstonia* (Rubiaceae). Evolutionary Ecology 26:909–926.

[CIT0033] Godsoe W, Larson MA, Glennon KL, Segraves KA 2013 Polyploidization in *Heuchera cylindrica* (Saxifragaceae) did not result in a shift in climatic requirements. American Journal of Botany 100:496–508. 23400493 10.3732/ajb.1200275

[CIT0034] Goldblatt P, Takei M 1993 Chromosome cytology of the African genus *Lapeirousia* (Iridaceae-Ixioideae). Annals of the Missouri Botanical Garden 80:961–973.

[CIT0035] Greilhuber J, Dolezel J, Lysák MA, Bennett MD 2005 The origin, evolution and proposed stabilization of the terms ‘genome size’ and ‘C-value’ to describe nuclear DNA contents. Annals of Botany 95:255–260. 15596473 10.1093/aob/mci019 PMC4246724

[CIT0036] Greilhuber J, Ebert I 1994 Genome size variation in *Pisum sativum*. Genome 37:646–655. 18470109 10.1139/g94-092

[CIT0106] Greilhuber J, Temsch EM, Loureiro J 2007 Nuclear DNA content measurement. In: Dolezel J, Greilhuber J, Suda J, eds. Flow cytometry with plant cells: analysis of genes, chromosomes and genomes. Weinheim: Wiley-VCH, 67–101.

[CIT0107] Gussone G 1832 Supplementum ad Florae Siculae Prodromum, vol. 1 Regia Typographia, Neapoli.

[CIT0039] Hamilton AP 1980 *Gladiolus* L. Flora Europaea 5:101–102.

[CIT0108] Hijmans RJ, van Etten J, Cheng J, Mattiuzzi M, Sumner M, Greenberg JA, Lamigueiro OP, Bevan A, Racine EB, Shortridge A, Ghosh A, 2017 Package ‘raster’ https://cran.r-project.org/package=raster. (21 May 2017).

[CIT0041] Hijmans RJ, Phillips S, Leathwick J, Elith J 2015 dismo: species distribution modeling. R package version 1.0-12. Vienna: The R Foundation for Statistical Computing http://cran.r-project.org. (21 May 2017).

[CIT0109] Hothorn T, Bretz F, Westfall P, Heiberger RM 2017 Multcomp: simultaneous inference for general linear hypotheses http://CRAN.Rproject.org/package=multcomp. (21 April 2016).

[CIT0043] Husband BC 2000 Constraints on polyploid evolution: a test of the minority cytotype exclusion principle. Proceedings of the Royal Society of London B: Biological Sciences 267:217–223. 10.1098/rspb.2000.0990 PMC1690524 10714875

[CIT0044] Husband BC 2004 The role of triploid hybrids in the evolutionary dynamics of mixed-ploidy populations. Biological Journal of the Linnean Society 82:537–546.

[CIT0045] Husband BC, Baldwin SJ, Sabara HA 2016 Direct vs. indirect effects of whole-genome duplication on prezygotic isolation in *Chamerion angustifolium*: implications for rapid speciation. American Journal of Botany 103:1259–1271. 27440792 10.3732/ajb.1600097

[CIT0046] Husband BC, Baldwin SJ, Suda J 2013 The incidence of polyploidy in natural plant populations: major patterns and evolutionary processes. In: Greilhuber J., Dolezel J., and Wendel J. F. eds. Plant genome diversity. Vienna: Springer Vienna, 255–276.

[CIT0047] Husband BC, Sabara HA 2004 Reproductive isolation between autotetraploids and their diploid progenitors in fireweed, *Chamerion angustifolium* (Onagraceae): research review. New Phytologist 161:703–713. 10.1046/j.1469-8137.2004.00998.x 33873724

[CIT0048] Husband BC, Schemske DW 1998 Cytotype distribution at a diploid-tetraploid contact zone in *Chamerion* (Epilobium) *angustifolium* (Onagraceae). American Journal of Botany 85:1688–1694. 21719413

[CIT0049] Husband BC, Schemske DW 2000 Ecological mechanisms of reproductive isolation between diploid and tetraploid *Chamerion angustifolium*. Journal of Ecology 88:689–701.

[CIT0050] Jersáková J, Castro S, Sonk N, Milchreit K, Schödelbauerová I, Tolasch T, Dötterl S 2010 Absence of pollinator-mediated premating barriers in mixed-ploidy populations of *Gymnadenia conopsea* sl (Orchidaceae). Evolutionary Ecology 24: 1199–1218.

[CIT0051] Jiao Y, Wickett NJ, Ayyampalayam S, Chanderbali AS, Landherr L, Ralph PE, Tomsho LP, Hu Y, Liang H, Soltis PS, Soltis DE, Clifton SW, Schlarbaum SE, Schuster SC, Ma H, Leebens-Mack J, dePamphilis CW 2011 Ancestral polyploidy in seed plants and angiosperms. Nature 473:97–100. 21478875 10.1038/nature09916

[CIT0052] Kao RH 2007 Asexuality and the coexistence of cytotypes. The New Phytologist 175:764–772. 17688591 10.1111/j.1469-8137.2007.02145.x

[CIT0053] Kamari G, Blanche C, Garbari F 2001 Mediterranean chromosome number reports: 11. Flora Mediterranea 11:435–483.

[CIT0054] Kennedy BF, Sabara HA, Haydon D, Husband BC 2006 Pollinator-mediated assortative mating in mixed ploidy populations of *Chamerion angustifolium* (Onagraceae). Oecologia 150: 398–408. 17024387 10.1007/s00442-006-0536-7

[CIT0055] Kolář F, Stech M, Trávnícek P, Rauchová J, Urfus T, Vít P, Kubesová M, Suda J 2009 Towards resolving the *Knautia arvensis* agg. (Dipsacaceae) puzzle: primary and secondary contact zones and ploidy segregation at landscape and microgeographic scales. Annals of Botany 103:963–974. 19196717 10.1093/aob/mcp016 PMC2707883

[CIT0056] Kron P, Suda J, Husband BC 2007 Applications of flow cytometry to evolutionary and population biology. Annual Review of Ecology, Evolution, and Systematics 38:847–876.

[CIT0057] Laport RG, Minckley RL, Ramsey J 2016 Ecological distributions, phenological isolation, and genetic structure in sympatric and parapatric populations of the *Larrea tridentata* polyploid complex. American Journal of Botany 103:1358–1374. 27440793 10.3732/ajb.1600105

[CIT0058] Levin DA 1975 Minority cytotype exclusion in local plant populations. Taxon 24:35–43.

[CIT0059] Levin DA 2002 The role of chromosomal change in plant evolution. Oxford: Oxford University Press.

[CIT0060] Lexer C, van Loo M 2006 Contact zones: natural labs for studying evolutionary transitions. Current Biology 16:R407–R409. 16753551 10.1016/j.cub.2006.05.007

[CIT0061] Loureiro J, Rodriguez E, Dolezel J, Santos C 2007 Two new nuclear isolation buffers for plant DNA flow cytometry: a test with 37 species. Annals of Botany 100:875–888. 17684025 10.1093/aob/mcm152 PMC2749623

[CIT0062] Löve A, Kjellqvist E 1973 Cytotaxonomy of Spanish plants. II. Monocotyledons. Lagascalia 7:147–182.

[CIT0063] Maceira NO, Haan AD, Lumaret R, Billon M, Delay J 1992 Production of 2n gametes in diploid subspecies of *Dactylis glomerata* L. 1. Occurrence and frequency of 2n pollen. Annals of Botany 69:335–343.

[CIT0064] Mallet J, Meyer A, Nosil P, Feder JL 2009 Space, sympatry and speciation. Journal of Evolutionary Biology 22:2332–2341. 19732264 10.1111/j.1420-9101.2009.01816.x

[CIT0065] Marques I, Loureiro J, Draper D, Castro M, Castro S 2017 How much do we know about the frequency of hybridization and polyploidy in the mediterranean region?Plant Biology. doi:10.1111/plb.12639. 10.1111/plb.12639 28963818

[CIT0066] Mifsud S, Hamilton AP 2013 Preliminary observations from long-term studies of *Gladiolus* L. (Iridaceae) for the Maltese Islands. Webbia 68:51–56.

[CIT0067] Monteiro-Henriques T, Martins MJ, Cerdeira JO, Silva PC, Arsénio P, Silva Á, Bellu A, Costa JC 2016 Bioclimatological mapping tackling uncertainty propagation: application to mainland Portugal. International Journal of Climatology 36:400–411.

[CIT0068] Muñoz‐Pajares AJ, Perfectti F, Loureiro J, Abdelaziz M, Biella P, Castro M, Castro S, Gómez JM 2017 Niche differences may explain the geographic distribution of cytotypes in *Erysimum mediohispanicum*. Plant Biology 20(Suppl. 1):139–147. 28741843 10.1111/plb.12605

[CIT0069] Münzbergová Z, Surinová M, Castro S 2013 Absence of gene flow between diploids and hexaploids of *Aster amellus* at multiple spatial scales. Heredity 110:123–130. 23169557 10.1038/hdy.2012.87 PMC3554448

[CIT0070] Nilsson O, Lassen P 1971 Chromosome numbers of vascular plants from Austria, Mallorca and Yugoslavia. Botaniska Notiser 124:270–276.

[CIT0071] Otto SP, Whitton J 2000 Polyploid incidence and evolution. Annual Review of Genetics 34:401–437. 10.1146/annurev.genet.34.1.401 11092833

[CIT0072] Pannell JR, Obbard DJ, Buggs RJ 2004 Polyploidy and the sexual system: what can we learn from *Mercurialis annua?* Biological Journal of the Linnean Society 82:547–560.

[CIT0073] Pérez E, Pastor Díaz JE 1994 Contribución al estudio cariológico de la familia Iridaceae en Andalucía Occidental. Lagascalia 17:257–272.

[CIT0074] Petit C, Bretagnolle F, Felber F 1999 Evolutionary consequences of diploid-polyploid hybrid zones in wild species. Trends in Ecology & Evolution 14:306–311. 10407427 10.1016/s0169-5347(99)01608-0

[CIT0075] Phillips SJ 2008 Transferability, sample selection bias, and background data in presence-only modelling: a response to Peterson *et al*. (2007). Ecography 31:272–278.

[CIT0076] Phillips SJ, Anderson RP, Schapire RE 2006 Maximum entropy modeling of species geographic distributions. Ecological Modelling 190:231–259.

[CIT0077] Queirós M 1979 Números cromossómicos para a flora Portuguesa: 16–37. Boletim da Sociedade Broteriana Série 2 53:15–28.

[CIT0078] Queirós M 1980 Números cromossómicos para a flora portuguesa: 38–63. Boletim da Sociedade Broteriana Série 2 54:47–64.

[CIT0079] R Core Development Team 2016 A language and environment for statistical computing. Vienna: R Foundation for Statistical Computing http://www.R-project.org/. (21 April 2016).

[CIT0080] Ramsey J 2007 Unreduced gametes and neopolyploids in natural populations of *Achillea borealis* (Asteraceae). Heredity 98:143–150. 17091127 10.1038/sj.hdy.6800912

[CIT0081] Ramsey J, Ramsey TS 2014 Ecological studies of polyploidy in the 100 years following its discovery. Philosophical Transactions of the Royal Society B: Biological Sciences. doi:10.1098/rstb.2013.0352. 10.1098/rstb.2013.0352 PMC4071525 24958925

[CIT0082] Ramsey J, Schemske DW 1998 Pathways, mechanisms, and rates of polyploid formation in flowering plants. Annual Review of Ecology and Systematics 29:467–501.

[CIT0083] Rodriguez DJ 1996 A model for the establishment of polyploidy in plants. The American Naturalist 147:33–46.

[CIT0084] Schoener TW 1970 Nonsynchronous spatial overlap of lizards in patchy habitats. Ecology 51:408–418.

[CIT0085] Segraves KA, Thompson JN 1999 Plant polyploidy and pollination: floral traits and insect visits to diploid and tetraploid *Heuchera grossulariifolia*. Evolution 53:1114–1127. 28565509 10.1111/j.1558-5646.1999.tb04526.x

[CIT0087] Soltis DE, Albert VA, Leebens-Mack J, Bell CD, Paterson AH, Zheng C, Sankoff D, Depamphilis CW, Wall PK, Soltis PS 2009 Polyploidy and angiosperm diversification. American Journal of Botany 96:336–348. 21628192 10.3732/ajb.0800079

[CIT0088] Soltis DE, Buggs RJ, Doyle JJ, Soltis PS 2010 What we still don’t know about polyploidy. Taxon 59:1387–1403.

[CIT0089] Soltis DE, Soltis PS 1999 Polyploidy: recurrent formation and genome evolution. Trends in Ecology & Evolution 14:348–352. 10441308 10.1016/s0169-5347(99)01638-9

[CIT0090] Ståhlberg D 2009 Habitat differentiation, hybridization and gene flow patterns in mixed populations of diploid and autotetraploid *Dactylorhiza maculata* s.l. (Orchidaceae). Evolutionary Ecology 23:295.

[CIT0091] Strid A, Franzen R 1981 In Chromosome number reports LXXIII. Taxon 30:829–842.

[CIT0092] Suda J, Kron P, Husband BC, Trávníček P 2007 Flow cytometry and ploidy: applications in plant systematics, ecology and evolutionary biology. In: Doležel J, Greilhuber J, Suda J, eds. Flow cytometry with plant cells: analysis of genes, chromosomes and genomes. Weinheim: Wiley-VCH, 103–130.

[CIT0093] Suda J, Oberlander KC, Dreyer LL 2013 Two new species of *Oxalis* (Oxalidaceae) from the Greater Cape Floristic Region. Phytotaxa 124:13–24.

[CIT0094] Susnik F, Lovka EH 1973 Reports. IOPB Chromosome numbers reports XLI. Taxon 22:462–463.

[CIT0095] Szczepaniak M, Kamiński R, Kuta E, Słomka A, Heise W, Cieślak E 2016 Natural hybridization between *Gladiolus palustris* and *G. imbricatus* inferred from morphological, molecular and reproductive evidence. Preslia 88:137–161.

[CIT0096] Thompson KA, Husband BC, Maherali H 2014 Climatic niche differences between diploid and tetraploid cytotypes of *Chamerion angustifolium* (Onagraceae). American Journal of Botany 101:1868–1875. 25366852 10.3732/ajb.1400184

[CIT0097] Thuiller W, Georges D, Engler R, Breiner F, Georges MD, Thuiller CW 2016 Package ‘biomod2’. https://cran.r-project.org/package=biomod2.

[CIT0098] Trávníček P, Kubátová B, Curn V, Rauchová J, Krajníková E, Jersáková J, Suda J 2010 Remarkable coexistence of multiple cytotypes of the *Gymnadenia conopsea* aggregate (the fragrant orchid): evidence from flow cytometry. Annals of Botany 107:77–87. 21059612 10.1093/aob/mcq217 PMC3002475

[CIT0099] Van Raamsdonk LWD, De Vries T 1989 Biosystematic studies in European species of *Gladiolus* (Iridaceae). Plant Systematics and Evolution 165:189–198.

[CIT0100] Visger CJ, Germain-Aubrey CC, Patel M, Sessa EB, Soltis PS, Soltis DE 2016 Niche divergence between diploid and autotetraploid *Tolmiea*. American Journal of Botany 103:1396–1406. 27507838 10.3732/ajb.1600130

[CIT0101] Warren DL, Glor RE, Turelli M 2008 Environmental niche equivalency versus conservatism: quantitative approaches to niche evolution. Evolution 62:2868–2883. 18752605 10.1111/j.1558-5646.2008.00482.x

[CIT0102] Wefferling KM, Castro S, Loureiro J, Castro M, Tavares D, Hoot SB 2017 Cytogeography of the subalpine marsh marigold polyploid complex (*Caltha leptosepala* s.l., Ranunculaceae). American Journal of Botany 104:271–285. 28183833 10.3732/ajb.1600365

[CIT0103] Wood TE, Takebayashi N, Barker MS, Mayrose I, Greenspoon PB, Rieseberg LH 2009 The frequency of polyploid speciation in vascular plants. Proceedings of the National Academy of Sciences 106:13875–13879. 10.1073/pnas.0811575106 PMC2728988 19667210

[CIT0104] Zozomová-Lihová J, Malánová-Krásná I, Vít P, Urfus T, Senko D, Svitok M, Kempa M, Marhold K 2015 Cytotype distribution patterns, ecological differentiation, and genetic structure in a diploid-tetraploid contact zone of *Cardamine amara*. American Journal of Botany 102:1380–1395. 26290560 10.3732/ajb.1500052

