# Supplementary material for: Complex cytogeographical patterns reveal a dynamic tetraploid–octoploid contact zone
Source: AoB Plants. 2018 Feb 14;10(2):ply012. doi: 10.1093/aobpla/ply012 (PMC5844219; doi:10.1093/aobpla/ply012)
Supplement: Supporting Information [file ply012_suppl_supporting_information.docx]

**SUPPORTING INFORMATION**

**Table S1** – Geographic information of sampled *Gladiolus* populations. ID code, DNA ploidy level, sample size and information about the location and geographical coordinates (angular) are given for each population. ID codes in bold represent populations with *Gladiolus communis* (*Gc*) and *Gladiolus italicus* (*Gi*) growing together. *Gladiolus communis* presented four cytotypes (4*x* – tetraploids, 6*x* – hexaploids, 8*x* – octoploids and 9*x* – nonaploids), while *G. italicus* presented only duodecaploid (12*x*) individuals. Populations marked with * represent populations used in chromosome counts.

| ID code | DNA Ploidy level (n) | | Location | Geographical coordinates | |
| --- | --- | --- | --- | --- | --- |
|  | Gc | Gi |  | Latitude | Longitude |
| MC153 | 4*x* (44) |  | Valverde, Santarém, Portugal | 39.45794 | -8.85369 |
| MC034 | 4*x* (26) |  | Amiais de Cima, Santarém, Portugal | 39.46061 | -8.75144 |
| MC037 | 4*x* (30) |  | Casais Monizes, Alcobaça, Portugal | 39.4608 | -8.89088 |
| MC150 | 4*x* (31) |  | Fonteinhas, Porto de Mós, Portugal | 39.4823 | -8.77042 |
| MC149 | 4*x* (35) |  | Cabeça das Pombas, Porto de Mós, Portugal | 39.49715 | -8.79206 |
| MC147 * | 4*x* (42) |  | Casal Duro, Fátima, Portugal | 39.57156 | -8.72667 |
| MC146 | 4*x* (3) | 12*x* (2) | Casal do Suão, Fátima, Portugal | 39.60689 | -8.71474 |
| DT11 | 4*x* (2) |  | Vila Velha de Rodão, Castelo Branco | 39.65356 | -7.69009 |
| DT10 | 4*x* (2) |  | Idanha-a-Nova, Castelo Branco, Portugal | 39.69477 | -7.78098 |
| MC222 | 4*x* (34) |  | Vale da Mua, Castelo Branco, Portugal | 39.69477 | -7.78098 |
| SC037 | 4*x* (13) |  | Barreiros, Leiria, Portugal | 39.7964 | -8.85395 |
| LM009 | 4*x* (24) |  | Valeirão, Pombal, Portugal | 39.8684 | -8.7611 |
| LM001 | 4*x* (24) |  | Casal Fernão João, Pombal, Portugal | 39.9158 | -8.65211 |
| LM006 | 4*x* (17) |  | Casal Fernão João, Pombal, Portugal | 39.91789 | -8.6562 |
| SC043 | 4*x* (35) |  | Antões, Pombal, Portugal | 39.96736 | -8.77196 |
| MC234 | 4*x* (30) |  | Moita do Boi, Pombal, Portugal | 39.97942 | -8.74266 |
| MC194 | 4*x* (32) |  | Castelhanas, Soure, Portugal | 39.98274 | -8.72756 |
| MC195 * | 4*x* (39) |  | Castelhanas, Soure, Portugal | 39.98665 | -8.72668 |
| SC044 | 4*x* (5) |  | Castelhanas, Soure, Portugal | 39.98744 | -8.74219 |
| MC197 | 4*x* (41) |  | Vascos, Soure, Portugal | 39.99569 | -8.67563 |
| MC192 | 4*x* (11) |  | Casal de Santo António, Soure, Portugal | 39.99724 | -8.74108 |
| MC203 | 4*x* (21) |  | Regateira, Góis, Portugal | 40.17067 | -8.10327 |
| MC202 | 4*x* (11) |  | Vale do Gueiro, Vila Nova de Poiares, Portugal | 40.20516 | -8.21416 |
| MC204 | 4*x* (21) |  | Casal de S. José, Arganil, Portugal | 40.20526 | -8.05387 |
| SC055 | 4*x* (35) |  | Secarias, Arganil, Portugal | 40.24508 | -8.03521 |
| JC001 | 4*x* (6) |  | Penacova, Penacova, Portugal | 40.26659 | -8.28116 |
| MC206 | 4*x* (22) |  | Vale do Matouco, Arganil, Portugal | 40.26881 | -8.1451 |
| MC205 | 4*x* (31) |  | Pousadouros, Tábua, Portugal | 40.26896 | -8.04568 |
| SC054 | 4*x* (13) |  | Coja, Arganil, Portugal | 40.27031 | -7.98444 |
| MC212 * | 4*x* (45) |  | Carapinha, Tábua, Portugal | 40.29412 | -8.09082 |
| JC009 | 4*x* (6) |  | São Pedro De Alva, Coimbra, Portugal | 40.30049 | -8.16638 |
| MC210 | 4*x* (8) |  | Covelo de Baixo, Tábua, Portugal | 40.32391 | -8.09137 |
| MC209 | 4*x* (22) |  | Ázere, Tábua, Portugal | 40.33529 | -8.09207 |
| MC208 | 4*x* (5) |  | Nagozela, Santa Comba Dão, Portugal | 40.45014 | -8.05349 |
| MC187 | 6*x* (29) |  | Carvalhal, Penacova, Portugal | 40.30821 | -8.19913 |
| MC211 * | 6*x* (26) |  | Covelo de Baixo, Tábua, Portugal | 40.3136 | -8.09344 |
| MC152 | 8*x* (4) | 12*x* (32) | Feiteira, Santarém, Portugal | 39.42256 | -8.83183 |
| MC151 | 8*x* (4) | 12*x* (23) | Coutada de Cima, Santarém, Portugal | 39.43799 | -8.7779 |
| MC154 | 8*x* (25) | 12*x* (13) | Casais do Chão de Mendiga, Porto de Mós, Portugal | 39.52468 | -8.85429 |
| MC032 * | 8*x* (30) |  | Alcaria, Fátima, Portugal | 39.56421 | -8.78816 |
| MC155 | 8*x* (9) | 12*x* (22) | Pragais, Porto de Mós, Portugal | 39.57958 | -8.81942 |
| MC145 | 8*x* (2) | 12*x* (6) | Milheirice, Fátima, Portugal | 39.62782 | -8.72085 |
| MC395 | 8*x* (24) |  | Santa Catarina da Serra, Fátima, Portugal | 39.6664 | -8.68534 |
| MC027 | 8*x* (21) |  | Santa Catarina da Serra, Fátima, Portugal | 39.67179 | -8.6821 |
| MC025 | 8*x* (3) |  | Ruge Água, Leiria, Portugal | 39.789 | -8.62085 |
| MC199 | 8*x* (36) |  | Vérigo, Soure, Portugal | 39.94442 | -8.57491 |
| MC198 | 8*x* (3) |  | Salgueiro, Soure, Portugal | 39.95214 | -8.58133 |
| MC200 | 8*x* (5) |  | Outeiro, Soure, Portugal | 39.97424 | -8.47408 |
| SC158 | 8*x* (5) |  | Vale de Poios, Pombal, Portugal | 39.97909 | -8.53983 |
| MC200A | 8*x* (8) |  | Ribeira de Alcalamouque, Soure, Portugal | 40.00444 | -8.45882 |
| MC190 * | 8*x* (53) |  | Casal da Rola, Soure, Portugal | 40.02146 | -8.71541 |
| SC045 | 8*x* (19) |  | Casal da Rola de Cima, Soure, Portugal | 40.02876 | -8.71737 |
| MC015 | 8*x* (28) | 12*x* (2) | Rabaçal, Penela, Portugal | 40.03225 | -8.43642 |
| MC370 | 8*x* (4) |  | Cavadas, Pombal, Portugal | 40.04072 | -8.75673 |
| MC013 | 8*x* (30) |  | Zambujal, Condeixa-a-Nova, Portugal | 40.05251 | -8.45642 |
| SC050 | 8*x* (27) |  | Camparca, Soure, Portugal | 40.05894 | -8.65606 |
| MC011 | 8*x* (24) |  | Casmilo, Condeixa-a-Nova, Portugal | 40.06095 | -8.50322 |
| MC374 | 8*x* (5) |  | Vales, Figueira da Foz, Portugal | 40.08155 | -8.80047 |
| MC006 | 8*x* (13) |  | Arrifana, Condeixa-a-Nova, Portugal | 40.08765 | -8.51106 |
| MC157 | 8*x* (3) | 12*x* (27) | Antanhol, Coimbra, Portugal | 40.1619 | -8.46086 |
| JC008 | 8*x* (9) |  | Marcos dos Pereiros, Coimbra, Portugal | 40.16665 | -8.41494 |
| SC002 | 8*x* (30) |  | Ferrugenta, Figueira da Foz, Portugal | 40.17705 | -8.82848 |
| SC033 | 8*x* (23) |  | Serra da Boa Viagem, Figueira da Foz, Portugal | 40.19532 | -8.89348 |
| MC144 | 8*x* (43) |  | Santo António dos Olivais, Coimbra, Portugal | 40.20853 | -8.40075 |
| MC143 * | 8*x* (38) |  | Relvinha, Coimbra, Portugal | 40.23889 | -8.43151 |
| MC142 | 8*x* (41) |  | Lôgo de Deus, Coimbra, Portugal | 40.26295 | -8.41314 |
| SC001 | 8*x* (29) |  | Brasfemes, Coimbra, Portugal | 40.26413 | -8.40991 |
| MC182 | 8*x* (30) |  | Trouxemil, Coimbra, Portugal | 40.27786 | -8.44533 |
| MC186 | 8*x* (23) |  | Telhado, Penacova, Portugal | 40.30728 | -8.33973 |
| MC188 | 8*x* (30) |  | Louredo, Penacova, Portugal | 40.35184 | -8.35776 |
| MC181 | 8*x* (30) |  | Mealhada, Coimbra, Portugal | 40.36726 | -8.45775 |
| MC189 | 8*x* (6) |  | Anadia, Aveiro, Portugal | 40.46232 | -8.43689 |
| MC213 | 4*x* (16), 6*x* (1) |  | Alcaria, Fátima, Portugal | 39.5637 | -8.77687 |
| JMC001 | 4*x* (6), 6*x* (2) |  | Secarias, Arganil, Portugal | 40.24689 | -8.03339 |
| MC148 | 4*x* (453), 8*x* (1) | 12*x* (3) | Carrascos, Fátima, Portugal | 39.54983 | -8.75783 |
| MC201 * | 4*x* (8), 8*x* (15) |  | Covelos, Coimbra, Portugal | 40.17012 | -8.28642 |
| MC207 | 4*x* (18), 8*x* (1) |  | Ázere, Tábua, Portugal | 40.34179 | -8.08996 |
| MC196 | 6*x* (1), 8*x* (20) |  | Borda do Rio, Soure, Portugal | 40.00512 | -8.70998 |
| MC191 | 6*x* (1), 8*x* (1) |  | Louriçal, Soure, Portugal | 40.00975 | -8.72784 |
| JC010 | 6*x* (4), 8*x* (14) |  | Luso, Aveiro, Portugal | 40.37578 | -8.37109 |
| MC193 * | 4*x* (1), 6*x* (4),8*x* (99), 9*x* (2) |  | Casal de Santo António, Soure, Portugal | 39.98978 | -8.73567 |
| MC159 | 4*x* (28) |  | Grândola, Grândola, Portugal | 38.20238 | -8.62507 |
| MC050 | 4*x* (2) |  | Algalé, Alcácer do Sal, Portugal | 38.27983 | -8.29287 |
| MC046 | 4*x* (13) |  | Santana, Sesimbra, Portugal | 38.45344 | -9.09083 |
| AM001 | 4*x* (16) |  | Paião, Montemor-o-Novo, Portugal | 38.60961 | -8.26568 |
| MC178 | 4*x* (33) |  | Alandroal, Alandroal, Portugal | 38.7058 | -7.40776 |
| MC041 | 4*x* (30) |  | Freixial, Loures, Portugal | 38.90263 | -9.15484 |
| MC039 | 4*x* (24) |  | Romão Charneca, Loures, Portugal | 38.90346 | -9.08455 |
| MC180 | 4*x* (35) |  | Vale da Seda, Fronteira, Portugal | 39.09706 | -7.68025 |
| MC158 | 4*x* (37) |  | Foz, Sever do Vouga, Portugal | 40.69068 | -8.41344 |
| MC250 | 4*x* (7) |  | Parada, Sever do Vouga, Portugal | 40.7701 | -8.2939 |
| MC302 | 4*x* (4) |  | Paredes, Vale de Cambra, Portugal | 40.81631 | -8.37471 |
| MC236 | 4*x* (11) |  | Agrela, Paços de Ferreira, Portugal | 41.2562 | -8.46647 |
| MC242 | 4*x* (3) |  | Santa Eulália, Vila Pouca de Aguiar, Portugal | 41.50069 | -7.79299 |
| MC240 | 6*x* (3) |  | Póvoa de Lenhoso, Braga, Portugal | 41.58447 | -8.32197 |
| MC168 | 8*x* (9) | 12*x* (24) | Budens, Vila do Bispo, Portugal | 37.08206 | -8.82402 |
| MC169 | 8*x* (30) |  | Vila Moura, Faro, Portugal | 37.09428 | -8.09808 |
| MC172 | 8*x* (2) | 12*x* (15) | Tavira, Faro, Portugal | 37.13949 | -7.65385 |
| MC162 | 8*x* (30) | 12*x* (1) | Colos, Ourique, Portugal | 37.71288 | -8.43586 |
| CS001 | 8*x* (3) | 12*x* (3) | Pinheiro, Aljustrel, Portugal | 37.89733 | -8.29525 |
| MC175 | 8*x* (1) | 12*x* (7) | Peso, Vidigueira, Portugal | 38.13894 | -7.67163 |
| MC223 | 8*x* (3) |  | Arronches, Portalegre, Portugal | 39.11088 | -7.25028 |
| MCD001 | 4*x* (5), 6*x* (1) |  | Fojo dos Morcegos, Arrábida, Portugal | 38.45607 | -9.01801 |
| MC232 | 4*x* (23), 6*x* (3) |  | Parada, Sever do Vouga, Portugal | 40.77817 | -8.29725 |
| MC173 | 4*x* (33), 8*x* (1) |  | Borracheira, Tavira, Portugal | 37.24766 | -7.69962 |
| MC164 | 4*x* (27), 8*x* (3) |  | Carvalho, Monchique, Portugal | 37.37825 | -8.5119 |
| MC176 | 4*x* (31), 8*x* (1) |  | Alqueva, Portel, Portugal | 38.19684 | -7.53172 |
| MC238 | 4*x* (5), 6*x* (2) |  | Chão, Vila Nova de Cerveira, Portugal | 41.92757 | -8.68125 |


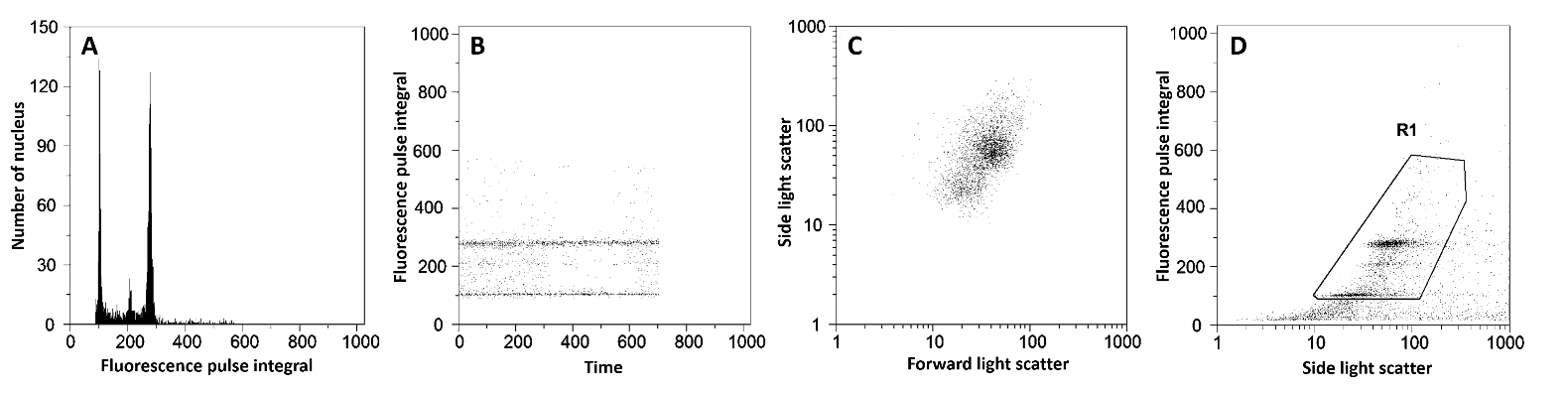
**Figure S1** – Flow cytometry graphics analyzed for each sample: A) fluorescence pulse integral in linear scale (FL); B) FL vs. time; C) forward light scatter (FS) vs. side light scatter (SS), both in logarithmic (log) scale; and D) FL vs. SS in log scale. R1 in D) presents a polygonal region that was applied to all the other graphics to digitally remove some of the debris.

**Table S2** – Genome size variation in *Gladiolus communis*. For each population and DNA ploidy level, the following values are given for the holoploid genome size (2C/pg): mean, standard deviation of the mean (SD), coefficient of variation (CV, %), minimum (Min) and maximum (Max). Sample size, Monoploid genome size (1Cx/pg) and reference standard used in the analyses [*Solanum lycopersicum* (*S.l.*) and *Pisum sativum* (*P.s.*)] are also given. Genome values are presente in picograms (pg). DNA ploidy levels: tetraploid (4*x*), hexaploid (6*x*), octoploid (8*x*) and nonaploid (9*x*).

| **ID code** | **DNA Ploidy level** |  | **Holoploid genome size (2C)** | | | | | |  | **Monoploid genome size (1Cx)** | **Reference standard** |
| --- | --- | --- | --- | --- | --- | --- | --- | --- | --- | --- | --- |
|  |  |  | **Mean** | **SD** | **CV (%)** | **Min** | **Max** | **N** |  |  |  |
| AM001 | 4*x* |  | 2.73 | 0.05 | 1.81 | 2.78 | 2.65 | 6 |  | 0.68 | *S.l.* |
| CS001 | 8*x* |  | 5.41 | - | - | - | - | 1 |  | 0.68 | *S.l.* |
| DT010 | 4*x* |  | 2.78 | 0.09 | 3.13 | 2.79 | 2.63 | 3 |  | 0.68 | *S.l.* |
| DT011 | 4*x* |  | 2.66 | 0.02 | 0.70 | 2.69 | 2.65 | 3 |  | 0.67 | *S.l.* |
| MC006 | 8*x* |  | 5.40 | 0.11 | 1.96 | 5.44 | 5.24 | 3 |  | 0.66 | *S.l.* |
| MC011 | 8*x* |  | 5.34 | 0.15 | 2.75 | 5.51 | 5.24 | 3 |  | 0.67 | *S.l.* |
| MC013 | 8*x* |  | 5.30 | 0.03 | 0.62 | 5.33 | 5.27 | 3 |  | 0.66 | *S.l.* |
| MC015 | 8*x* |  | 5.34 | 0.04 | 0.70 | 5.36 | 5.31 | 2 |  | 0.64 | *S.l.* |
| MC025 | 8*x* |  | 5.31 | 0.03 | 0.01 | 5.34 | 5.27 | 3 |  | 0.66 | *S.l.* |
| MC027 | 8*x* |  | 5.45 | 0.10 | 0.02 | 5.54 | 5.35 | 3 |  | 0.68 | *S.l.* |
| MC032 | 8*x* |  | 5.57 | 0.09 | 0.02 | 5.62 | 5.47 | 3 |  | 0.70 | *S.l.* |
| MC034 | 4*x* |  | 2.68 | 0.03 | 0.01 | 2.70 | 2.64 | 3 |  | 0.67 | *S.l.* |
| MC037 | 4*x* |  | 2.68 | 0.01 | 0.00 | 2.69 | 2.67 | 3 |  | 0.67 | *S.l.* |
| MC039 | 4*x* |  | 2.69 | 0.03 | 0.01 | 2.72 | 2.67 | 3 |  | 0.67 | *S.l.* |
| MC041 | 4*x* |  | 2.66 | 0.02 | 0.01 | 2.69 | 2.64 | 3 |  | 0.67 | *S.l.* |
| MC046 | 4*x* |  | 2.74 | 0.04 | 0.02 | 2.78 | 2.70 | 3 |  | 0.68 | *S.l.* |
| MC050 | 4*x* |  | 2.74 | 0.03 | 0.01 | 2.76 | 2.71 | 2 |  | 0.68 | *S.l.* |
| MC144 | 8*x* |  | 5.21 | - | - | - | - | 1 |  | 0.65 | *S.l.* |
| MC147 | 4*x* |  | 2.67 | - | - | - | - | 1 |  | 0.67 | *S.l.* |
| MC148 | 4*x* |  | 2.66 | 0.06 | 2.14 | 2.76 | 2.62 | 5 |  | 0.66 | *S.l.* |
|  | 8*x* |  | 5.30 | - | - | - | - | 1 |  | 0.66 | *S.l.* |
| MC151 | 8*x* |  | 5.30 | - | - | - | - | 1 |  | 0.66 | *S.l.* |
| MC152 | 8*x* |  | 5.30 | - | - | - | - | 1 |  | 0.66 | *S.l.* |
| MC154 | 8*x* |  | 5.52 | 0.09 | 1.61 | 5.66 | 5.26 | 18 |  | 0.69 | *S.l.* |
| MC193 | 4*x* |  | 2.86 | - | - | - | - | 1 |  | 0.71 | *S.l.* |
|  | 6*x* |  | 4.07 | 0.03 | 0.01 | 4.09 | 4.02 | 4 |  | 0.68 | *S.l.* |
|  | 8*x* |  | 5.39 | 0.15 | 0.03 | 5.69 | 5.13 | 30 |  | 0.67 | *S.l.* |
|  | 9*x* |  | 6.10 | 0.18 | 0.03 | 6.23 | 5.98 | 2 |  | 0.68 | *S.l.* |
| MC201 | 4*x* |  | 2.62 | 0.01 | 0.00 | 2.62 | 2.61 | 2 |  | 0.65 | *S.l.* |
|  | 8*x* |  | 5.38 | 0.12 | 0.02 | 5.51 | 5.30 | 3 |  | 0.67 | *S.l.* |
| MC232 | 4*x* |  | 2.67 | 0.07 | 0.02 | 2.78 | 2.58 | 18 |  | 0.67 | *S.l.* |
|  | 6x |  | 4.01 | 0.07 | 0.02 | 4.06 | 3.93 | 3 |  | 0.67 | *S.l.* |
| MC238 | 4*x* |  | 2.71 | 0.02 | 0.01 | 2.73 | 2.67 | 5 |  | 0.68 | *S.l.* |
|  | 6*x* |  | 4.16 | 0.04 | 0.01 | 4.19 | 4.13 | 2 |  | 0.69 | *S.l.* |
| M302 | 4*x* |  | 2.78 | - | - | - | - | 1 |  | 0.69 | *S.l.* |
| MC370 | 8*x* |  | 5.54 | 0.15 | 0.03 | 5.73 | 5.38 | 4 |  | 0.69 | *P.s.* |
| MC395 | 8*x* |  | 5.59 | 0.04 | 0.01 | 5.61 | 5.54 | 3 |  | 0.70 | *S.l.* |
| SC001 | 8*x* |  | 5.41 | 0.08 | 0.01 | 5.51 | 5.35 | 3 |  | 0.68 | *S.l.* |
| SC002 | 8*x* |  | 5.34 | 0.04 | 0.01 | 5.39 | 5.30 | 3 |  | 0.67 | *S.l.* |
| SC298 | 4*x* |  | 2.86 | 0.03 | 0.01 | 2.89 | 2.83 | 3 |  | 0.71 | *P.s.* |

**Table S3** – Mixed-ploidy populations of *Gladiolus communis*. For each population, the total number of analyzed individuals (N total) and percentage of each cytotype within the population are presented. Populations are identified by ID codes following Appendix 1 and are organized in groups according with their cytotype composition (4*x* + 6*x*, 4*x* + 6*x* + 8*x* + 9*x*, 4*x* + 8*x*, and 6*x* + 8*x*). DNA ploidy levels: tetraploids (4*x*), hexaploid (6*x*), octoploid (8*x*) and nonaploid (9*x*). Populations where all the individuals were sampled are underlined (results in Figure 3).

| **Mixed-ploidy populations** | **N total** |  | **Cytotypes (%)** | | | |
| --- | --- | --- | --- | --- | --- | --- |
|  |  |  | **4*x*** | **6*x*** | **8*x*** | **9*x*** |
| **4*x* + 6*x*** |  |  |  |  |  |  |
| JMC001 | 8 |  | 75.0 | 25.0 |  |  |
| MC213 | 17 |  | 94.1 | 5.9 |  |  |
| MC232 | 26 |  | 88.5 | 11.5 |  |  |
| MC238 | 7 |  | 71.4 | 28.6 |  |  |
| MCD001 | 6 |  | 83.3 | 16.7 |  |  |
| **4*x* + 6*x* + 8*x* + 9*x*** |  |  |  |  |  |  |
| MC193 | 106 |  | 0.9 | 3.8 | 93.4 | 1.9 |
| **4*x* + 8*x*** |  |  |  |  |  |  |
| MC148 | 449 |  | 99.8 |  | 0.2 |  |
| MC164 | 30 |  | 90.0 |  | 10.0 |  |
| MC173 | 34 |  | 97.1 |  | 2.9 |  |
| MC176 | 32 |  | 96.9 |  | 3.1 |  |
| MC201 | 23 |  | 34.8 |  | 65.2 |  |
| MC207 | 19 |  | 94.7 |  | 5.3 |  |
| **6*x* + 8*x*** |  |  |  |  |  |  |
| JC010 | 18 |  |  | 22.2 | 77.8 |  |
| MC191 | 2 |  |  | 50.0 | 50.0 |  |
| MC196 | 21 |  |  | 4.8 | 95.2 |  |

**Table S4** – DNA ploidy levels of the offspring of pure- and mixed-ploidy populations of *Gladiolus communis*. For each population, the total number of seeds analyzed (N total) and percentage of each DNA ploidy level within the offspring are presented. DNA ploidy levels: tetraploids (4*x*), pentaploid (5*x*), hexaploid (6*x*), octoploid (8*x*) and aneuploid (An.).

| **Populations** | **N total** |  | **Offspring DNA ploidy level (%)** | | | | |
| --- | --- | --- | --- | --- | --- | --- | --- |
|  |  |  | **4*x*** | **5*x*** | **6*x*** | **8*x*** | **An.** |
| **Pure-ploidy** |  |  |  |  |  |  | 0.96 |
| 4*x* | 515 |  | 99.04 |  |  |  | 0.96 |
| 6*x* | 264 |  |  | 20.47 | 17.80 |  | 61.73 |
| 8*x* | 540 |  |  |  |  | 100.00 |  |
| **Mixed-ploidy** |  |  |  |  |  |  |  |
| 4*x* | 70 |  | 100.00 |  |  |  |  |
| 8*x* | 60 |  |  |  |  | 100.00 |  |
